# Sediment disturbance by Ediacaran bulldozers and the roots of the Cambrian explosion

**DOI:** 10.1038/s41598-018-22859-9

**Published:** 2018-03-14

**Authors:** Luis A. Buatois, John Almond, M. Gabriela Mángano, Sören Jensen, Gerard J. B. Germs

**Affiliations:** 1 Department of Geological Sciences, University of Saskatchewan, 114 Science Place, Saskatoon, Saskatchewan S7N 5E2 Canada; 2 Natura Viva cc, Cape Town, 8001 South Africa; 3 Área de Paleontología, Facultad de Ciencias, Universidad de Extremadura, E-06006 Badajoz, Spain; 4 Department of Geology, University of the Free State, Bloemfontein, South Africa

## Abstract

Trace fossils of sediment bulldozers are documented from terminal Ediacaran strata of the Nama Group in Namibia, where they occur in the Spitskop Member of the Urusis Formation (Schwarzrand Subgroup). They consist of unilobate to bilobate horizontal to subhorizontal trace fossils describing scribbles, circles and, more rarely, open spirals and meanders, and displaying an internal structure indicative of active fill. Their presence suggests that exploitation of the shallow infaunal ecospace by relatively large bilaterians was already well underway at the dawn of the Phanerozoic. Efficient burrowing suggests coelom development most likely linked to metazoan body-size increase. These trace fossils are the earliest clear representatives so far recorded of sediment bulldozing, an activity that may have had a negative impact on suspension-feeding and/or osmotroph communities, as well as on matgrounds, representing early examples of ecosystem engineering and trophic-group amensalism. The occurrence of sediment bulldozers may have promoted the establishment of gradients in horizontal and vertical distribution of organic material in connection with spatially heterogeneous environments on the sea floor at a critical time in Earth evolution.

## Introduction

Ediacaran benthic marine ecosystems were dominated by microbial mats that sealed the sediment from the water column essentially in the absence of bioturbation, and which have been referred to as matgrounds^1^. Ediacaran ichnofaunas are typically dominated by surficial to very shallow, non-specialized horizontal trails^2–5^. These trails are interpreted as produced by vagile bilaterian metazoans that are thought to exploit organic matter concentrated within microbial mats. Other less common, and more controversial, components of Ediacaran ichnofaunas are paired scratch marks associated with the enigmatic body fossil *Kimberella*, which has been traditionally assigned to a molluscan-grade producer^3,6–9^. However, it has been noted that no unequivocal derived molluscan features are present in *Kimberella*^10^, and even its assignment to bilaterians has been considered equivocal^11^. Dickinsoniid trace fossils, suggesting external digestion of microbial mats, are also known^7,8,12–14^; an alternative interpretation involving passive transport^15^ is less likely^12,16^.

The demise of extensive microbial mats formed in normal marine settings and the concomitant disappearance of matground-related lifestyles as a consequence of increased sediment mixing by burrowing animals have been referred to as the Cambrian agronomic revolution^1^. In turn, the notion of a Cambrian substrate revolution highlights the evolutionary and ecological effects of substrate changes on benthic metazoans^17,18^. The shift from matgrounds to mixgrounds is thought to have been signalled by an increase in the spatial complexity and heterogeneity of marine environments^19,20^. However, the impact of this evolutionary event is still controversial, with some authors emphasizing its significance for sediment mixing and ecosystem engineering^1,21–23^ and others interpreting that bioturbation did not play a major role until later in the Palaeozoic^24,25^. Also, ongoing studies suggest that patchiness in the distribution of organisms may have been significant in the Ediacaran as well^26,27^.

**Figure 1:**
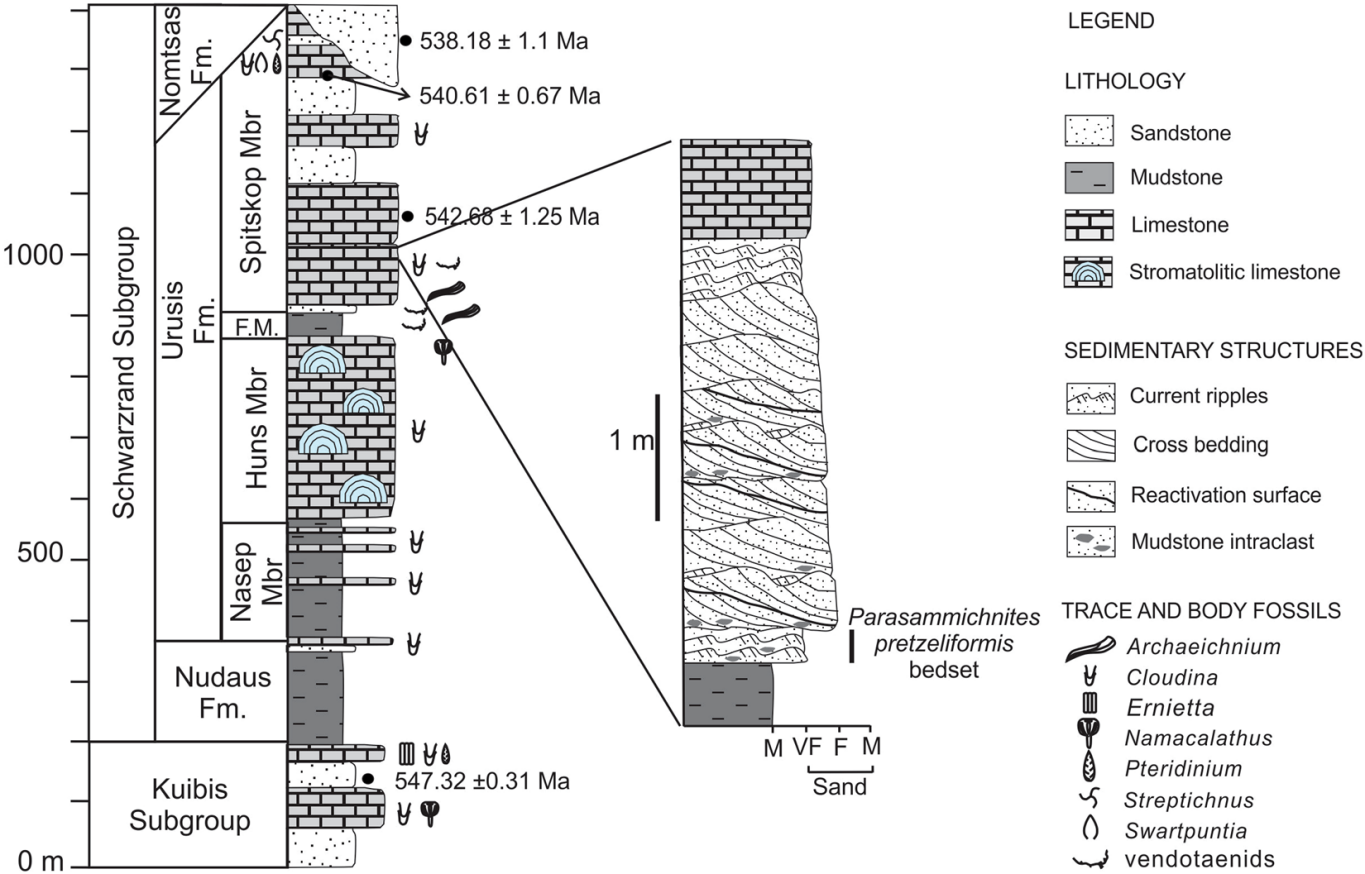
Stratigraphic framework. Synthetic stratigraphy of the Nama Group in the Witputs sub-basin^41^ and detailed stratigraphic section measured 1.2 km ESE of the Koelkrans camp in Fish River Canyon/Gondwana Canyon Park region (sections designed and drawn by Luis A. Buatois using Corel DRAW X4 software). FM = Feldschuhhorn Member. Radiometric ages after refs^33,35^. The 547+/−0.3 age is from the Zaris sub-basin. Thickness of Spitskop Member is less than 6 m in the study area. Carbonate packages in the main Nama section (based on the more complete, thicker basinal succession to the west) include siliciclastic intervals, especially towards the cratonic areas to the east.

The search for key evidence of changing animal-substrate interactions during this critical time in the evolution of Earth’s biosphere is therefore of great importance. In particular, sediment bulldozers – moderately large (*circa* 1 cm wide or larger) vagile, deposit-feeding animals that displace substantial volumes of sediment while burrowing within the sediment^28,29^ would have been particularly effective in promoting such major changes in benthic ecology. Sediment bulldozers backfill their own tunnels using the material dug out in front of the body and transported to the posterior area, typically (but not exclusively) producing wide and flat trace fossils^30^. It is generally believed that these bulldozers are absent in Ediacaran and lowermost Cambrian rocks, represented by the *Treptichnus pedum* Zone, and that they first appear in the overlying *Rusophycus avalonensis* Zone, where they are accompanied by bilobed arthropod trace fossils that may have had a significant effect on incipient sediment-mixing. The Ediacaran–Fortunian Nama Group of Namibia, with its thick, laterally continuous, virtually undeformed and relatively well-dated shallow-marine successions, is ideally suited for this search. In this paper we document the presence of moderately abundant, almost 1 cm wide, trace fossils produced by sediment bulldozers in shallow-marine deposits of terminal Ediacaran age in the Spitskop Member of the Urusis Formation (Schwarzrand Subgroup, Nama Group) (Fig. 1) exposed in the Fish River Canyon region of southern Namibia (SI Appendix, Fig. S1). This finding demonstrates that colonisation of the shallow infaunal ecospace by moderately large metazoans, resulting in concomitant sediment disturbance, was already underway during the Ediacaran–Cambrian transition, representing a prelude to Cambrian-style bioturbation.

## Stratigraphic Setting

There are no radiometric dates for the level of the basal Cambrian GSSP on Newfoundland, but it is currently considered to be 541 ± 1 Ma^31^. Historically the Ediacaran–Cambrian boundary in southern Namibia has been placed within an unconformity at the base of the Nomtsas Formation (Schwarzrand Subgroup, Nama Group), with pronounced valley incision that locally has eroded deep into sediments of the underlying Urusis Formation^32–34^. This initial location of the boundary was consistent with ash beds, from the lower and upper part of the Spitskop Member (Urusis Formation, Schwarzrand Subgroup), from which zircons had originally been dated to 545.1 ± 1 Ma and 543.3 ± 1 Ma respectively^33^. However, recalibration of the Spitskop radiometric data indicates revised dates of 542.68 ± 1.25 Ma (terminal Ediacaran) and 540.61 ± 0.67 Ma (within error of the Ediacaran–Cambrian boundary), respectively^35^. An ash bed from the Hoogland Member towards the base of the Nama Group (Zaris Formation, Kuibus Subgroup) has yielded an age of 547.4 ± 0.3 Ma^33^^,^^36^, now slightly modified to 547.32 ± 0.31 Ma^35^. The lower part of the Nomtsas Formation has yielded an age of 539.4 ± 1 Ma, recently recalibrated to 538.18 ± 1.11 Ma^35^. A negative excursion in carbon isotopes within carbonates above the highest recorded *Cloudina* in Oman^37^ has been dated to around 541 Ma. This prominent negative excursion in carbon isotopes, recognized globally at a level close to the Ediacaran–Cambrian boundary, has not been detected in the Nama Group. However, carbon isotopes in carbonates of the Schwarzrand Group are close to +2 per mil PDB, similar to values found in latest Ediacaran successions in China and Oman^33,35^. The available radiometric and carbon isotope data therefore concur that the Ediacaran–Cambrian boundary in the Nama Group is situated below the Nomtsas incision surface, and probably within the uppermost part of the Spitskop Member.

Palaeontological evidence for this placement of the boundary is the presence in various outcrops of the Kuibis and Schwarzrand subgroups of the early skeletal fossils *Cloudina* and *Namacalathus* (commonly recorded in terminal Ediacaran strata) as well as Ediacara-type fossils, including *Pteridinium* and *Swartpuntia* in the upper part of the Spitskop Member in Swartpunt Farm^38^. The branching burrow *Streptichnus*, which occurs in the upper part of the Spitskop Member^39^ but above the *Pteridinium* beds also in Swartpunt Farm, is now within error of the Ediacaran-Cambrian transition, and may in fact be regarded as a representative of Cambrian-style bioturbation. The Nomtsas Formation contains unambiguous examples of the Cambrian-type trace fossil *Treptichnus pedum* in various sections, most notably in Sonntagsbrunn Farm^34,36,40^.

A detailed stratigraphic framework for the Nama Group^41,42^ allows for correlation of the Urusis Formation across the Witputs subbasin in southern Namibia (SI Appendix, Fig. S2). The easternmost outcrops included in these correlations are exposed southeast of Feldschuhhorn, approximately 25 km west of our study area, representing deposition towards the landward margin. Depositional sequence D includes the Feldschuhhorn Member and the lower part of the Spitskop Member, whereas depositional sequence E (Sequence 5) consists of the upper part of the Spitskop Member^41^. In places (e.g. Koedolaagte), both sequences have been removed by erosion due to incision of the Nomtsas Formation (SI Appendix, Fig. S2). Similarly on Sonntagsbrunn Farm, some 5 km west of the study area, only thin remnants of the Spitskop Member are locally present. Fallen blocks suggest the original presence here of a thicker Spitskop Member that has otherwise been truncated by two episodes of valley incision, subsequently infilled by the Nomtsas Formation, which also locally truncated the Feldschuhorn Member^34,42^. Different stratigraphic architectures for the Urusis Formation on both sides (southwest-northeast) of the Nomtsas valley incision have been depicted^41,42^. However, detailed mapping in the area indicates that the Huns, Feldschuhhorn and Spitskop members are laterally persistent following a west-east trend^43^.

Integration of regional information and local stratigraphy indicates that the trace-fossil beds in the northern Fish River Canyon area occur in the clastic interval present in the lower half of the Spitskop Member within depositional sequence D^41^. The Spitskop Member records deposition in a mixed carbonate-siliciclastic platform. Regionally, carbonate facies are dominated by stromatolitic limestone, cross-bedded calcarenite, domal bioherms and pinnacle reefs encompassing from the mid shelf to the carbonate slope^41^. Carbonate deposits interfinger landwards with offshore, offshore-transition and shoreface sandstone^41,42,44^. Shoreface intervals in the Urusis Formation commonly contain evidence of oscillatory flows, such as hummocky cross stratification and wave ripples, the latter showing crests oriented northwest-southeast, parallel to the palaeoshoreline^41,42^. However, evidence of tidal influence has been noted as well^41^ and, therefore, some of these sandstone units may be better referred to as tidal beaches or tidally modulated shorefaces^45,46^. This is consistent with our sedimentological analysis of ichnofossil-bearing beds in the Koelkrans area of the Fish River Canyon, which indicates a tidal origin for the sandstone deposits here (see SI Appendix). The absence of structures indicative of oscillatory flows and the dominance of tidal structures in these proximal deposits may suggest deposition in a more protected embayment, where tidal effects were amplified and open marine waves buffered.

Placement of the trace fossil-bearing beds within the Spitskop Member (as opposed to a younger, early Cambrian succession) is further supported on palaeontological grounds. The Spitskop and Feldschuhhorn member beds at Koelkrans campsite, just a few hundred meters west of the new trace-fossil locality, contain abundant vendotaenids, such as *Vendotaenia antiqua* (SI Appendix, Fig. S3), identical to those described from the Feldschuhhorn Member in Sonntagsbrunn farm^47^. In addition, the Spitskop beds in the Koelkrans area contain the tubular body fossil *Archaeichnium*, which is typically recorded in association with iconic members of the Ediacara biota elsewhere^48–50^. The beds at Koelkrans campsite can be easily traced laterally (within walking distance) to the ichnofossil locality, providing further support for its stratigraphic position.

Even assuming strong diachronism within the Urusis Formation, the ichnofossil-bearing deposits clearly occur below the subaerial unconformity at the base of the Nomtsas Formation (SI Appendix, Fig. S4), constraining the stratigraphic position of the trace fossils analysed. The 542.68 Ma date from the lower part of the Spitskop Member at Farm Swartpunt argues in favour of a terminal Ediacaran age for the trace-fossil bearing beds.

The trace fossil-bearing beds occur within a sandy clastic interval of the Spitskop Member that passes abruptly upward into thin-bedded Spitskop Member carbonates recording a major deepening episode^41,42^. In the Fish River Canyon study area, the Spitskop carbonates in turn are sharply overlain by the Nomtsas Formation, which consists of massive, thick-bedded, fine-grained sandstone, followed by medium-bedded, ripple cross-laminated, fine- to very-fine grained sandstone and a heterolithic sandstone-siltstone interval (SI Appendix, Fig. S5). In the study section there is no evidence of palaeovalley incision beneath the – here broadly planar – basal Nomtsas unconformity such as has been documented in other areas of the basin^34,42^, indicating that the Koelkrans succession was formed in an interfluve area. However, the Spitskop Member at the trace fossil site is very thin (less than 6 m). This is attributed to significant erosion even in interfluve areas as a result of sediment bypass during the sea level fall that took place in the basin during the earliest Fortunian combined with general pinch out of the carbonate belt in a landward direction, a feature that has been long recognized in stratigraphic analysis of the Nama Group^41,43,44^.

### Trace-fossil occurrence

**Figure 2:**
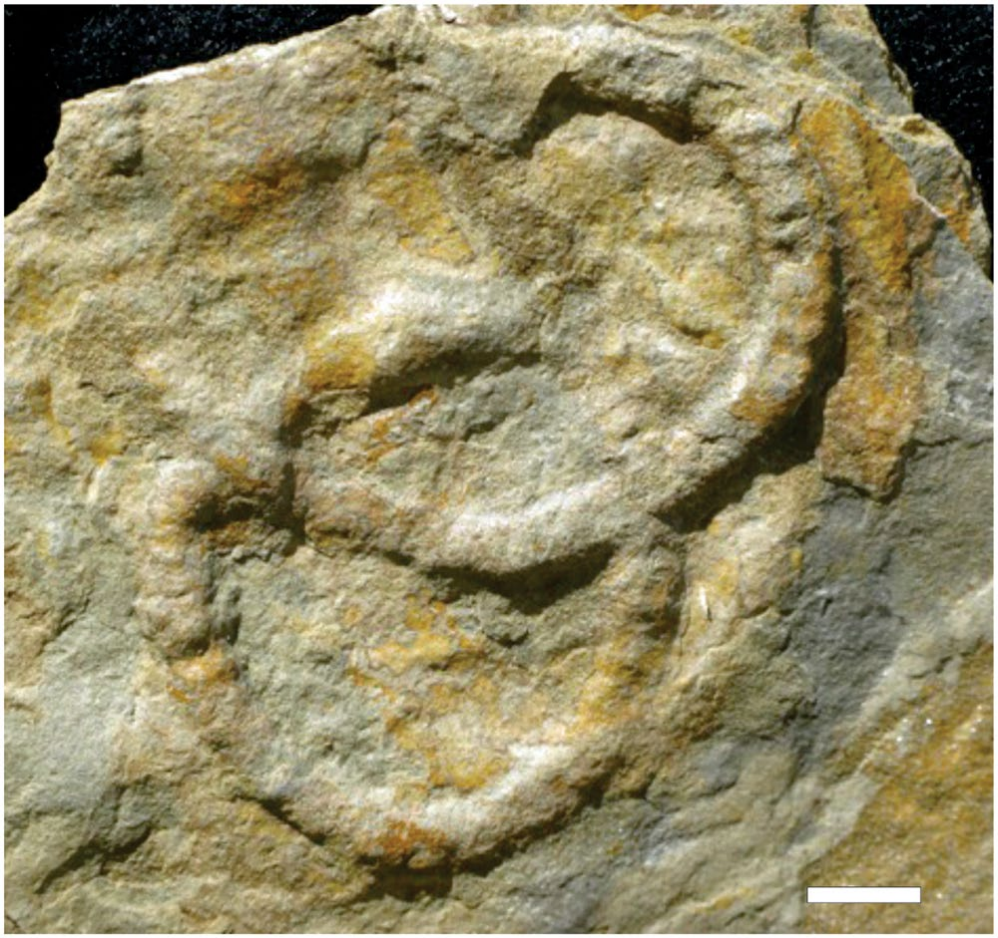
Slab with holotype of *Parapsammichnites pretzeliformis* from the Urusis Formation. Scale bar is 1 cm.

**Figure 3:**
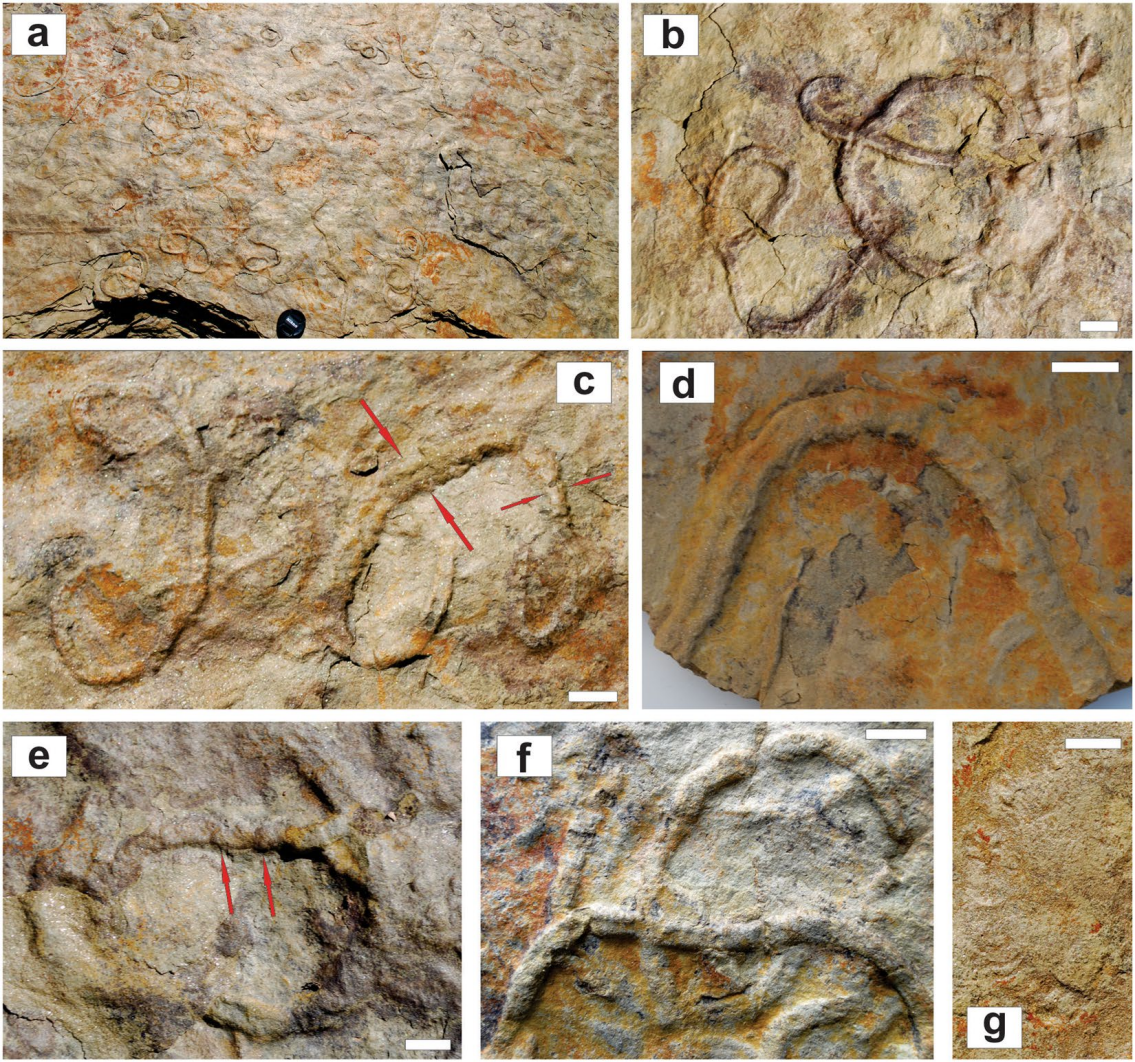
Morphological variability of *Parapsammichnites pretzeliformis* n. igen. and n. isp. from the Urusis Formation. **(a)** General view of sandstone base showing moderate density of horizontal trace fossils depicting scribbles, circles and spirals. Lens cover is 5.5 cm wide. **(b)** Close-up of scribbles, showing bilobate nature of the structures. **(c)** Trace fossil displaying self-overcrossing and pretzel shape. Long trace fossil displaying several self-overcrossings and significant width changes along the course (large and short arrows). Large arrows show expansion “envelope” of reworked sediment. **(d)** Unilobate trace fossil showing sediment pads and grading into a bilobate segment (left). **(e)** Specimens displaying inclined arcuate ridges (sediment pads), resulting locally in constricted aspect. Arrows mark arcuate sediment pads slightly offset from the axis. **(f)** Several specimens showing transitions between unilobate to bilobate segments. **(g)** Weathered specimen displaying meniscate laminae, hinting towards packing in sediment pads. All views are from sandstone bed soles. All scale bars are 2 cm long.

*Parapsammichnites pretzeliformis* n. igen. and n. isp. (SI Appendix) consists of unilobate to bilobate, actively infilled, horizontal to subhorizontal trace fossils describing scribbles, simple circles and, more rarely, open spirals and meanders (Figs 2, 3a–g, S6, S8a,b, S9a–g). Trace fossils are preserved as full-relief structures typically at the bases of 0.5–1 cm thick, current-ripple cross-laminated, micaceous, very fine-grained silty sandstone beds and, more rarely, on sandstone tops. Density of trace fossils is moderate to locally relatively high, with structures covering up to 14.1% of sandstone surfaces. Trace fossils are not evenly distributed on large bedding surfaces, but tend to show higher densities in certain areas, resulting in a heterogeneous, patchy distribution. Intensity of bioturbation as seen in cross-section is variable reaching up to 22.9% disturbance of the primary fabric (Fig. S7a,b).

Sandstone bases are locally covered by tool marks, primary current lineation and intraclasts (SI Appendix, Fig. S5a), indicating frequent, small-scale erosive events. Highly regular, comb-like tool marks ascribed to current-entrained vendobionts are common in this unit (SI Appendix, Fig. S5b). Syneresis cracks are locally present (SI Appendix, Fig. S5g). The trace fossils cross-cut the inorganic tool marks, indicating that they are post-depositional and penetrate from the top of the sandstone layer. Sandstone layers are 1–2 cm thick and stacked forming a 30 cm-thick bedset. These deposits are interpreted as the bottomsets of a shallow-subtidal dune complex (SI Appendix, Fig. S5c–f).

### Evidence of trace-fossil nature and attribution to a bilaterian tracemaker

In contrast to the usual debates surrounding Ediacaran trace fossils and problematic forms^16^, there is uncontroversial evidence that the structures analysed here are trace fossils of bilaterians. The bilaterian trace-fossil nature of these structures is indicated by: (1) heterogeneous (i.e. patchy) distribution on stratigraphic surfaces reflecting the ability to detect and imperfectly exploit an area enriched in organics; (2) active backfill and sediment displacement as illustrated by an envelope zone; (3) consistent width along the same toponomic level; (4) course characterised by scribbles, nested loops, pretzel-like forms and common self-overcrossing; (5) lack of preferential orientation (which would otherwise indicate current alignment of body fossils or tool marks made by entrained organisms); and (6) absence of frayed or angular terminations^51^.

In short, all morphological features of *Parapsammichnites pretzeliformis* indicate it was produced by a bilaterian. Giant protists are known to produce locomotion trails^52^, but these structures are morphologically simple and typically short. Experiments with modern actinians and platyhelminths that creep by cilia on mucus ribbons indicate that they may produce trails having lateral raised margins^53^. Some actinians may even produce trails displaying diffuse crescentic ridges^54^. However, these simple structures lack the internal complexity of *Parapsammichnites*, in particular the meniscate backfill locally organized in sediment pads and the bilobate structure. Equally important in supporting the bilaterian origin, there is no evidence that actinians or platyhelminths may produce scribbles, spirals or pretzel-like pathway patterns.

The general pattern of the excavations analysed is strongly reminiscent of a group of relatively large trace fossils with global distribution during the early Cambrian showing looping, circling, and self-overcrossing, including “*Taphrhelminthopsis*” *circularis*^55^ and *Psammichnites gigas*^30^. A characteristic feature of these Cambrian forms is that they are commonly preserved as full reliefs or positive epireliefs on sandstone tops and they reveal a complex internal structure involving a thin axial groove (typically the first feature to be lost by weathering) and a laminated backfill structure. The axial groove, locally with a sinusoidal development, has been interpreted as reflecting the passing of a snorkel-like device and prompted attribution to molluscs^30,56^. In any case, disregarding detailed morphological differences, affinities with these ichnotaxa are based on a similar way of exploiting the sediment and exploring substrate heterogeneities, clearly supporting a bilaterian origin and continuity of the “*Psammichnites* evolutionary lineage” across the Ediacaran-Cambrian transition^55^.

## Discussion

Because of the controversial affinities of most Ediacara-type body fossils, ichnological information is of paramount importance to track the earliest evidence of bilaterian organisms in the Neoproterozoic^57^. Claims of an earlier appearance of bioturbators representing ecosystem engineers^58^ based on the presence of supposed meniscate backfill structures in *Nenoxites*, remain controversial^16,59^. The structures consist of serially repeated modules, identical to those in problematic body fossils such as *Helanoichnus*, *Palaeopascichnus* and *Shaanxilithes*, arguing against a trace-fossil origin^16,59,60^.

Although global diversity and morphological complexity of Ediacaran ichnofaunas is low^23^, it is becoming clear that more sophisticated behavioural strategies (i.e. branching burrow systems) had appeared by the terminal Ediacaran in shallow-water settings^39,61^. *Parapsammichnites pretzeliformis* is distinguished from these forms, namely *Streptichnus narbonnei*^39^ and the so-called treptichnids^61^, by the absence of branching. The latter also distinguishes the trace fossils documented in this study from *Treptichnus pedum*, extensively recorded in the same area in lower Cambrian rocks^34^. From a palaeoenvironmental standpoint, all these more complex trace fossils of the Ediacaran-Cambrian transition occur broadly in a similar depositional belt, situated around the fair-weather wave base, either right above, under shallow subtidal conditions, or right below, in offshore transition to upper offshore settings.

The backfilled scribbling trace fossils documented here indicate the onset of a new style of burrowing exploration that indicates the presence of a coelom, whose origin was presumably associated with body-size increase^11,62–64^. This new ichnotaxon records a significant innovation in burrowing style at the Ediacaran–Cambrian transition, namely sediment bulldozing by metazoans of a size comparable to that of Cambrian animals. Body size is significant in biomechanics and physiology, representing a constraint on morphological complexity^62–64^, as well as playing a major role in ecosystem functioning^65^. The exploratory pathway pattern of *Parapsammichnites* indicates sediment reworking and processing of sediment common in Phanerozoic pascichnial structures, but essentially absent in Ediacaran simple trails and burrows, which typically display very simple patterns and only very rarely may show a tendency to form crude spirals^55,66^. Other factors being equal, sediment disturbance is directly linked to the cross-sectional area of the bioturbator^29^. In addition, it has been indicated that mouth size is also important in increasing sediment reworking because small mouths tend to be associated to selective ingestion of small volumes or organic-rich detritus^29^. Although any discussion of mouth size of the producer of *Parapsammichnites* would be completely speculative, we note that the Ediacaran–Cambrian transition has been regarded as a time of significant evolution in metazoan mouthparts, albeit in connection with predation rather than deposit feeding^67^.

In modern environments, burrowing deposit feeders tend to impact negatively on immobile suspension feeders to the point of excluding the affected group, illustrating a phenomenon referred to as trophic-group amensalism^68^ and representing an example of ecosystem engineering^69^. The resultant physical instability directly inhibits newly settled suspension feeding larvae, preventing attachment of sessile epifauna and facilitating remobilization of sediment that may clog the filtering devices of suspension feeders^68^. In particular, sediment bulldozing involves the forcible pushing-aside of sediment with the animal’s body or the manipulation of packages of sediment with appendages^30^. Sediment bulldozers are of paramount importance in ecosystem function^70,71^. Bulldozing by deposit feeders, as well as by scavengers and predators, is commonly conducive to remobilization of fine-grained particles and faeces, fluidization of muddy substrates, disruption of matgrounds, clogging of suspension-feeding systems, deepening of the redox discontinuity surface, and creation of dynamic heterogeneous environments, among other aspects^1,21,23,28,29,68^. On the other hand, bioturbators, through promoting transformations of the habitat, represent a dynamic force in the self-structuring and function of marine ecosystems^72^.

Integration of tools from benthic ecology and evolutionary palaeoecology has allowed better evaluation of behavioural diversification, ecospace occupation and ecosystem engineering in the colonisation of new habitats by providing a consistent conceptual framework of analysis^73–75^. Following these approaches, the Namibia trace fossils represent colonisation by a shallow-infaunal, highly mobile, deposit-feeder, moving through the sediment in a conveyor belt fashion, backfilling and transporting sediment. This style of sediment disturbance has not been previously documented convincingly from Ediacaran deposits. Although the density of trace fossils on the here described bedding plane is moderate by Phanerozoic standards, it is high if compared with other Ediacaran occurrences, which tend to consist of very low density suites of simple grazing trails^4,16^, but see^76^ for an exception. Earlier models imply a more or less gradual increase in the size of bioturbators since the beginning of the Phanerozoic^29^. However, the trace fossils documented herein demonstrate that an increase in size of bulldozing trace fossils to widths of 5–10 mm had already started by the terminal Ediacaran (SI Appendix, Figs S10 and S11).

There is strong interest in the underlying causes of the disappearance of the Ediacara biota, which may have involved the closing of the matground taphonomic window, a mass extinction of Ediacaran organisms, or biotic replacement^77^. The presence in the Fortunian of matgrounds associated with evidence of bilaterian activity in the absence, or great scarcity, of elements of the Ediacara biota has been taken as evidence of the persistence of this particular taphonomic window into the Phanerozoic, thereby arguing against the taphonomic hypothesis^78^. A recent study in the Spitskop Member at Farm Swartpunt reveals lower diversity in the local Nama Assemblage than in older assemblages, arguing against a catastrophic extinction event and in favour of a more gradual disappearance^79^. It has been therefore proposed that the gradual decline and final disappearance of the Ediacara biota were driven by increasing levels of competition with the newly evolving bilaterians^77,79–81^, although a more positive relationship has also been suggested^10^.

The co-occurrence of trace and body fossils in the same Ediacaran facies belts suggests that bilaterians and Ediacarans probably co-existed for almost 20 My without the former impacting negatively on the latter^16,23,26,57,82,83^. The presence of *Parapsammichnites pretzeliformis* in the Spitskop Member suggests that it is not the onset of bilaterians *per se* that should be deemed responsible for the decline of the Ediacara biota, but the appearance of bilaterians capable of bioturbation and sediment disturbance^16^. Efficient burrowing at a significant scale may have precipitated the final demise of diverse Ediacaran epifaunal communities. The majority of Ediacara soft-bodied biotas are thought to have been dominated by immobile organisms, some of which may have formed tiered epifaunal suspension-feeding communities^84^. Bulldozer feeding and defecation by vagile metazoans may have generated particle resuspension, destabilizing the substrate inhabited by a sessile epifauna and clogging their filtering devices. Although filtering devices have not been described from the Ediacara biota, functional morphology analysis combined with the study of fluid flow patterns suggests passive suspension feeding for some forms^85^. Alternatively, rangeomorphs and erniettomorphs have been interpreted as feeding through direct nutrient absorption (“osmotrophy”)^86^. In either case, sediment bulldozing may have negatively impacted on these communities. Although immobile suspension feeders were abundant and diverse in Cambrian soft substrates, this trophic group is remarkably absent in Fortunian strata. In fact, fabrics dominated by high densities of dwelling burrows of infaunal suspension feeders (e.g. *Skolithos* piperock) are known since Cambrian Stage 2 shallow-marine deposits, but are unknown in older strata^23^. Similar exclusion of immobile suspension feeders by bulldozers has been invoked as a macroevolutionary driver later in the Phanerozoic^28,29^. More specifically, the gradual demise of sessile suspension-feeding communities throughout the Phanerozoic has been attributed to the increased diversification of deposit feeders^28,29,87^. The Nama occurrence probably represents one of the earliest examples of ecosystem engineering and trophic-group amensalism^88^.

While evidence of matgrounds is extensive through most of the Ediacaran strata included in the Nama Group, there is a notable absence of structures indicative of microbial mats in the Koelkrans ichnofossil-bearing siliciclastic deposits. Although it may be argued that different depositional environments may have been involved, matgrounds in the Nama occur in similar nearshore heterolithic and sandstone-dominated deposits, such as those exposed in the Koelkrans area, arguing against a facies control. This fact is consistent with the notion that increased substrate disruption by infaunal organisms was conducive to the demise of matground-dominated ecosystems. However, abundant comb-like vendobiont-type tool marks in these beds suggest that elements of the Ediacara biota may have been present in nearby settings.

In addition, the occurrence of sediment bulldozers in subtidal settings must have stimulated the establishment of new gradients in horizontal and vertical distribution of organic material, thereby promoting spatially heterogeneity on the sea floor. Increasingly heterogeneous environments may have been conducive to more sophisticated foraging patterns and development of macroscopic sense organs in mobile bilaterians, allowing exploitation of patchily distributed resources^19,20^. The overall rarity of trace fossils attributed to sediment bulldozers in terminal Ediacaran rocks suggests that their ecological impact may have been limited and raises the issue of potential diachronism in evolutionary innovations across the Ediacaran-Cambrian transition. This is in line with the persistence of matgrounds in the Fortunian^78^ and the observation that primary sedimentary fabrics show very little disturbance in Fortunian strata^23,89,90^. In any case, the well-documented presence of trace fossils produced by infaunal bilaterians in the Ediacaran–Cambrian transition of the Nama Group clearly shows that the evolutionary lineages and behaviours that led to the more dramatic shifts in substrate properties later during the Phanerozoic were already present by the end of the Ediacaran.

## Materials and Methods

This study was based on an integration of sedimentological, stratigraphic and ichnological datasets. A bed-by-bed sedimentological log was measured in the succession containing the trace fossils. Conventional facies analysis was performed and beds were characterised in terms of lithology, physical sedimentary structures, bed contacts, and bed geometry. Bedform dimensions were measured at all scales. Interpretations in terms of depositional processes and sedimentary environments were based on parameters assessed during facies analysis. The succession was placed into a broader stratigraphic framework following regional mapping and correlation. The latter was based on the application of sequence stratigraphic tools, including recognition and delineation of depositional sequences, systems tracts, parasequences and surfaces of alostratigraphic importance. Trace fossils were described according to standard ichnological practice, which included the use of the concept of ichnotaxobases, distinctive morphological features of a trace fossil that display significant and readily detectable variability^46,91^. Measurements were taken of a number of parameters, such as trace fossil width and penetration depth. Density of trace fossils on bedding planes was assessed after estimating a percentage of covered surface using Adobe Photoshop and ImageJ softwares^92^.

## Electronic supplementary material

Supplementary Information

## References

[1] Seilacher A (1999). Biomat-related lifestyles in the Precambrian. Palaios.

[2] Gehling JG (1999). Microbial mats in terminal Proterozoic siliciclastic Ediacaran death masks. Palaios.

[3] Seilacher A, Buatois LA, Mángano MG (2005). Trace fossils in the Ediacaran–Cambrian transition: Behavioural diversification, ecological turnover and environmental shift. Palaeogeogr. Palaeoclimatol. Palaeoecol..

[4] Jensen, S., Droser, M. L. & Gehling, J. G. In Neoproterozoic geobiology and paleobiology (eds Kaufman, J. & Xiao, S.). *Topics in Geobiology* **27**, 115–157 (Springer, 2006).

[5] Buatois, L. A. & Mángano, M. G. In Microbial mats in siliciclastic depositional systems through time (eds Noffke, N. & Chafetz, H.). *SEPM Special Publication* **101**, 15–28 (2012).

[6] Fedonkin MA (2003). Origin of the metazoa in the light of Proterozoic fossil records. Paleont. Res..

[7] Gehling, J. G., Droser, M., Jensen, S. & Runnegar, B. In *Evolving Form and Function: Fossils and Development* (ed. Briggs, D.E.G.) 43–66 (Yale University, New Haven, 2005).

[8] Ivantsov AY (2013). Trace fossils of Precambrian metazoans “Vendobionta” and “Mollusks”. Strat. Geol. Correl..

[9] Gehling JG, Runnegar BN, Droser ML (2014). Scratch traces of large Ediacara bilaterian animals. J. Paleontol..

[10] Budd GE, Jensen S (2017). The origin of the animals and a ‘Savannah’hypothesis for early bilaterian evolution. Biol. Rev..

[11] Budd GE, Jensen S (2000). A critical reappraisal of the fossil record of the bilaterian phyla. Biol. Rev..

[12] Sperling EA, Vinther J (2010). A placozoan affinity for *Dickinsonia* and the evolution of late Proterozoic metazoan feeding modes. Evol. Develop..

[13] Ivantsov AY (2011). Feeding traces of proarticulata—the Vendian metazoa. Paleontol. J..

[14] Ivantsov AY, Malakhovskaya YE (2003). Giant traces of Vendian animals. Dokl. Earth Sci..

[15] McIlroy D, Brasier MD, Lang AS (2009). Smothering of microbial mats by macrobiota: Implications for the Ediacara biota. J. Geol. Soc..

[16] Buatois, L. A. & Mángano, M. G. In The trace-fossil record of major evolutionary events, vol. 1: Precambrian and Paleozoic (eds Mángano, M. G. & Buatois, L. A.). *Topics in Geobiology* **39**, 27–72 (Springer, 2016).

[17] Bottjer DJ, Hagadorn JW, Dornbos SQ (2000). The Cambrian substrate revolution. GSA Today.

[18] Mángano MG, Buatois LA (2017). The Cambrian revolutions: Trace-fossil record, timing, links and geobiological impact. Earth-Sci. Rev..

[19] Plotnick RE, Dornbos SQ, Chen J (2010). Information landscapes and sensory ecology of the Cambrian Radiation. Paleobiology.

[20] Mángano MG (2012). Nonbiomineralized carapaces in Cambrian seafloor landscapes (Sirius Passet, Greenland): Opening a new window into early Phanerozoic benthic ecology. Geology.

[21] Meysman FJ, Middelburg JJ, Heip CH (2006). Bioturbation: a fresh look at Darwin’s last idea. Trends Ecol. Evol..

[22] Erwin DH, Tweedt SM (2012). Ecological drivers of the Ediacaran–Cambrian diversification of Metazoa. Evol. Ecol..

[23] Mángano MG, Buatois LA (2014). Decoupling of body-plan diversification and ecological structuring during the Ediacaran-Cambrian transition: Evolutionary and geobiological feedbacks. P. Roy. Soc. B-Biol. Sci..

[24] Tarhan LG, Droser ML (2014). Widespread delayed mixing in early to middle Cambrian marine shelfal settings. Palaeogeogr. Palaeoclimatol. Palaeoecol..

[25] Tarhan LG, Droser ML, Planavsky NJ, Johnston DT (2015). Protracted development of bioturbation through the early Palaeozoic Era. Nature Geosci..

[26] Droser ML, Gehling JG (2015). The advent of animals: the view from the Ediacaran. Proc. Natl. Acad. Sci. USA.

[27] Finnegan, S., Droser, M. L. & Gehling, J. G. Unusual patchiness of Ediacaran benthic assemblages relative to Phanerozoic and modern benthic assemblages. Geological Society of America *Abstracts with Programs* **49**(6), (2017).

[28] Thayer, C. W. In *Biotic Interactions in Recent and Fossil Benthic* Communities (eds Tevesz, M. J. S. & McCall, P. L.) 479–625 (Plenum, New York, 1983).

[29] Thayer CW (1979). Biological bulldozers and the evolution of marine benthic communities. Science.

[30] Seilacher, A. *Trace Fossil Analysis*. 1–226 (Springer-Verlag, 2007).

[31] Peng, S., Babcock, L. E. & Cooper, R. A. In *The Geological Time Scale*, *Volume 2* (eds Gradstein, F. M., Ogg. J. G., Schmitz, M. D. & Ogg, G. M.) 437-488 (Elsevier, Amsterdam, 2012).

[32] Germs GJB (1972). New shelly fossils from Nam**a** Group, south-westAfrica. Am. J. Sci..

[33] Grotzinger JP, Bowring SA, Saylor BZ, Kaufman AJ (1995). Biostratigraphic and geochronologic constraints on early animal evolution. Science.

[34] Wilson JP (2012). Deep-water incised valley deposits at the Proterozoic-Cambrian boundary in southern Namibia contain abundant *Treptichnus pedum*. Palaios.

[35] Schmitz, M. D. In *The Geologic Time Scale*, *Volume 2* (eds Gradstein, F. M., Ogg. J. G., Schmitz, M. D. & Ogg, G. M.) 1045–1082 (Elsevier, Amsterdam, 2012).

[36] Geyer, G. & Uchman, A. Ichnofossil assemblages from the Nama Group (Neoproterozoic–Lower Cambrian) in Namibia and the Proterozoic–Cambrian boundary problem revisited. *Beringeria Spec*. *Iss*. **2**, 175–202 (1995).

[37] Bowring SA (2007). Geochronologic constraints on the chronostratigraphic framework of the Neoproterozoic Huqf Supergroup, Sultanate of Oman. Am. J. Sci..

[38] Narbonne GM, Saylor BZ, Grotzinger JP (1997). The youngest Ediacaran fossils from Southern Africa. J. Paleontol..

[39] Jensen S, Runnegar BN (2005). A complex trace fossil from the Spitskop Member (terminal Ediacaran–? Lower Cambrian) of southern Namibia. Geol. Mag..

[40] Crimes TP, Germs GJB (1982). Trace fossils from the Nama Group (Precambrian–Cambrian) of southwest Africa (Namibia). J. Paleontol..

[41] Saylor BZ (2003). Sequence stratigraphy and carbonate-siliciclastic mixing in a terminal Proterozoic foreland basin, Urusis Formation, Nama Group, Namibia. J. Sed. Res..

[42] Germs, G. J. B. In Evolution of the Damara Orogen of south west Africa/Namibia (ed. Miller, R. Mc. G.). *Geol*. *Soc*. *S*. *Afr*. *Spec*. *Publ*. **11**, 89–114 (1983).

[43] Germs, G. J. B., Miller, R. Mc. G., Frimmel, H. E. & Gaucher, C. In Neoproterozoic-Cambrian tectonics, global change and evolution: a focus on southwestern Gondwana (eds Gaucher, C., Sial, A. N., Halverson, G. P. & Frimmel, H. E.). *Develop*. *Precambrian Geol*. **16**, 183–203 (Elsevier, 2010).

[44] Saylor BZ, Grotzinger JP, Germs GJB (1995). Sequence stratigraphy and sedimentology of the Neoproterozoic Kuibis and Schwarzrand subgroups (Nama Group), southwestern Namibia. Precambrian Res..

[45] Dashtgard SE, Gingras MK, MacEachern JA (2009). Tidally modulated shorefaces. J. Sed. Res..

[46] Buatois, L. A. & Mángano, M. G. Ichnolo*gy*: Org*anism-substrate Interactions in Space and Time*. 1–358 (Cambridge University Press, 2011).

[47] Cohen PA (2009). Tubular compression fossils from the Ediacaran Nama Group, Namibia. J. Paleontol..

[48] Glaessner MF (1978). Re-examination of *Archaeichnium*, a fossil from the Nama Group. Ann. S. Afr. Mus..

[49] Hagadorn JW, Waggoner B (2000). Ediacaran fossils from the southwestern Great Basin, United States. J. Paleontol..

[50] Gehling JG, Droser ML (2013). How well do fossil assemblages of the Ediacara Biota tell time?. Geology.

[51] Droser, M. L., Gehling, J. G. & Jensen, S. In *Evolving Form and Function: Fossils and Development* (ed. Briggs, D.E.G.) 125–138 (Yale University, New Haven, 2005).

[52] Matz MV, Frank TM, Marshall NJ, Widder EA, Johnsen S (2008). Giant deep-sea protist produces bilaterian-like traces. Curr. Biol..

[53] Collins AG, Lipps JH, Valentine JW (2000). Modern mucociliary creeping trails and the bodyplans of Neoproterozoic trace-makers. Paleobiology.

[54] Liu AG, Mcllroy D, Brasier MD (2010). First evidence for locomotion in the Ediacara biota from the 565 Ma Mistaken Point Formation, Newfoundland. Geology.

[55] Jensen S (2003). The Proterozoic and earliest Cambrian trace fossil record: Patterns, problems and perspectives. Int. Comp. Biol..

[56] Seilacher A, Hagadorn JW (2010). Early molluscan evolution: Evidence from the trace fossil record. Palaios.

[57] Seilacher A (1989). Vendozoa: Organismic construction in the Proterozoic biosphere. Lethaia.

[58] Rogov V (2012). The oldest evidence of bioturbation on Earth. Geology.

[59] Brasier MD, McIlroy D, Liu AG, Antcliffe JB, Menon LR (2013). The oldest evidence of bioturbation on Earth: Comment. Geology.

[60] Meyer M, Schiffbauer JD, Xiao S, Cai Y, Hua H (2012). Taphonomy of the upper Ediacaran enigmatic ribbonlike fossil *Shaanxilithes*. Palaios.

[61] Jensen S, Saylor BZ, Gehling JG, Germs GJB (2000). Complex trace fossils from the terminal Proterozoic of Namibia. Geology.

[62] Valentine, J. W. *On the Origin of Phyla*. 1–608 (Chicago University Press, 2004).

[63] Schmidt-Nielsen, K. *Scaling*: *Why is Animal Size so Important?* 1–256 (Cambridge University Press, 1984).

[64] Koehl, M. A. R. In *Scaling in Biology* (eds Brown, J. H. & West, G. B.) 67–86 (Oxford University Press, New York, 2000).

[65] Emerson, M. C. In *Marine Biodiversity and Ecosystem Functioning: Frameworks*, *Methodologies*, *and Integration* (eds Solan, M., Aspden, R. J. & Paterson, D. M.) 85–100 (Oxford University Press, Oxford, 2012).

[66] Carbone C, Narbonne GM (2014). When life got smart: the evolution of behavioral complexity through the Ediacaran and early Cambrian of NW Canada. J. Paleontol..

[67] Klug C, Frey L, Pohle A, De Baets K, Korn D (2017). Palaeozoic evolution of animal mouthparts. Bull. Geosci..

[68] Rhoads DC, Young DK (1970). The influence of deposit-feeding organisms on sediment stability and community trophic structures. J. Mar. Res..

[69] Jones CG, Lawton JH, Shachak M (1994). Organisms as ecosystem engineers. Oikos.

[70] Solan M (2004). Extinction and ecosystem function in the marine benthos. Science.

[71] Solan, M., Scott, F., Dulvy, N. K., Godbold, J. A. & Parker, R. In *Marine Biodiversity and Ecosystem Functioning: Frameworks*, *Methodologies*, *and Integration* (eds Solan, M., Aspden, R. J. & Paterson, D. M.) 127–148 (Oxford University Press, Oxford, 2012).

[72] Lohrer AM, Thrush SF, Gibbs MM (2004). Bioturbators enhance ecosystem function through complex biogeochemical interactions. Nature.

[73] Minter, N., Buatois, L. A. & Mángano, M. G. In The trace-fossil record of major evolutionary events, vol. 1: Precambrian and Paleozoic (eds Mángano, M. G. & Buatois, L. A.). *Topics in Geobiology* **39**, 1–26 (Springer, 2016).

[74] Minter NJ (2017). Early bursts of diversification defined the faunal colonization of land. Nature Ecol. Evol..

[75] Bush, A. M., Bambach, R. K. & Erwin, D. H. In *Quantifying the Evolution of Early Life* (eds Laflamme, M., Schiffbauer, J. D. & Dornbos, S. Q.) 111–133 (Springer Netherlands, 2011).

[76] Meyer M (2014). Interactions between Ediacaran animals and microbial mats: Insights from Lamonte trevallis, a new trace fossil from the Dengying Formation of South China. Palaeogeogr. Palaeoclimatol. Palaeoecol..

[77] Laflamme M, Darroch SAF, Tweedt SM, Peterson KJ, Erwin DH (2013). The end of the Ediacara biota: Extinction, biotic replacement, or Cheshire Cat?. Gondwana Res..

[78] Buatois LA, Narbonne GM, Mángano MG, Carmona NB, Myrow P (2014). Relict ecosystems at the dawn of the Phanerozoic revolution. Nature Commun..

[79] Darroch SA (2015). Biotic replacement and mass extinction of the Ediacara biota. Proc. R. Soc. B.

[80] Darroch SA (2016). A mixed Ediacaran-metazoan assemblage from the Zaris Sub-basin, Namibia. Palaeogeogr. Palaeoclimatol. Palaeoecol..

[81] Schiffbauer JD (2016). The latest Ediacaran Wormworld fauna: Setting the ecological stage for the Cambrian Explosion. GSA Today.

[82] Bottjer, D. J. & Clapham, M. E. In *Neoproterozoic Geobiology and Paleobiology* (eds Xiao, S. & Kaufman, A. J.) 91–114 (Springer Netherlands, 2006).

[83] Droser ML, Tarhan LG, Gehling JG (2017). The rise of animals in a changing environment: global ecological innovation in the late Ediacaran. Annu. Rev. Earth Planet. Sci..

[84] Clapham ME, Narbonne GM (2002). Ediacaran epifaunal tiering. Geology.

[85] Rahman IA, Darroch SA, Racicot RA, Laflamme M (2015). Suspension feeding in the enigmatic Ediacaran organism *Tribrachidium* demonstrates complexity of Neoproterozoic ecosystems. Sci. Adv..

[86] Laflamme M, Xiao S, Kowalewski M (2009). Osmotrophy in modular Ediacara organisms. Proc. Natl. Acad. Sci. USA.

[87] Larson, D. W. & Rhoads, D. C. In *Biotic Interactions in Recent and Fossil Benthic* Communities (eds Tevesz, M. J. S. & McCall, P. L.) 627–648 (Springer US, 1983).

[88] Rhoads DC, Young DK (1970). The influence of deposit feeding organisms on sediment stability and community trophic structure. J. Mar. Res..

[89] Droser ML, Jensen S, Gehling JG, Myrow PM, Narbonne GM (2002). Lowermost Cambrian ichnofabrics from the Chapel Island Formation, Newfoundland: Implications for Cambrian substrates. Palaios.

[90] Droser ML, Jensen S, Gehling JG (2002). Trace fossils and substrates of the terminal Proterozoic–Cambrian transition: Implications for the record of early bilaterians and sediment mixing. Proc. Natl. Acad. Sci. USA.

[91] Bromley, R. G. *Trace Fossils: Biology*, *Taphonomy and Applications*. 1–361 (Chapman & Hall, 1996).

[92] Cao YM, Curran AH, Glumac B (2015). Testing the use of photoshop and imageJ for evaluating ichnofabrics. Geological Society of America. Abstracts with Programs.

