# Supplementary material for: Sediment disturbance by Ediacaran bulldozers and the roots of the Cambrian explosion
Source: Sci Rep. 2018 Mar 14;8:4514. doi: 10.1038/s41598-018-22859-9 (PMC5852133; doi:10.1038/s41598-018-22859-9)
Supplement: Supplementary file 1 — Supplementary Information [file 41598_2018_22859_MOESM1_ESM.pdf]

Supplementary Materials for

Sediment disturbance by Ediacaran bulldozers and the roots of the Cambrian explosion

Luis A. Buatois[1]*, John Almond[2], M. Gabriela Mángano[1], Sören Jensen[3] & Gerard J.B. Germs[4]

*To whom correspondence should be addressed. E-mail: luis.buatois@usask.ca

This PDF file includes:

Text S1-4

Figures S1–11

References

List of supplementary materials:

Text S1. Previous work on the ichnology of the Nama Group and the placement of the Ediacaran–Cambrian boundary

Text S2. Systematic description

Text S3. Ichnologic Remarks

Text S4. Sedimentology of the trace fossil-bearing deposits

Figure S1. Location maps

Figure S2. Stratigraphic cross-section of the Nama Group

Figure S3. The vendotaenid *Vendotaenia antiqua* in the Spitskop Member

Figure S4. General view of the studied outcrop

Figure S5. Sedimentological features

Figure S6. Polished sections of slabs containing *Parapsammichnites pretzeliformis*

Figure S7. Cross-section views of *Parapsammichnites pretzeliformis*

Figure S8. Density of *Parapsammichnites pretzeliformis* on bedding surfaces.

Figure S9. Morphological variability of *Parapsammichnites pretzeliformis*

Figure S10. Histogram showing size distribution of *Parapsammichnites pretzeliformis*

Figure S11. Changes in size of bulldozer-type trace fossils, as a proxy for body-size of the producers, across the Ediacaran–Cambrian transition

References

1. Previous work on the ichnology of the Nama Group and the placement of the Ediacaran–Cambrian boundary

Various studies have documented trace fossils in the Nama Group[32, 39, 40, 36, 61, 78, 93-95]. Trace fossils with evidence for sediment displacement, including forms or *Archaeonassa*-type, occur in the Vingerbreek Member of the Nudaus Formation[Ref. 40, plate 2:8; Ref. 94, fig 6B]. Trace fossils showing repeated vertical probes, aligning them to *Treptichnus*, occur in the Huns Member, Urusis Formation[61, 93]. Trace fossils somewhat similar to *Treptichnus pedum* have been mentioned in the Nasep Member, Urusis Formation[36]. The complex branched trace fossil *Streptichnus narbonnei* occurs within the Spitskop Member near the top of the Urusis Formation, in strata younger than 540.61 +/- 0.67 Ma[39], therefore within error of the Ediacaran–Cambrian transition. Under the assumption that there is no significant diachronism within the lithostratigraphic units, the *Archaeonassa*-type trace fossils from the Vingerbreek Member and the treptichnids from the Huns Member are older than 542.68 +/- 1.25 Ma and younger than 547.32 +/- 0.31 Ma. Structures resembling spreiten burrows and compared with *Zoophycos* have been recently recorded from the Zaris Formation[95]. However, the illustrated specimen does not have the morphologic features (e.g., internal structure, marginal tube) that are characteristic of *Zoophycos*[16]. Additionally, the occurrence of a single specimen prevents evaluation of its recurrence and makes interpretation of this structure as a trace fossil highly problematic[16]. Possible *Conichnus* have been recorded in the Schwarzrand Group[39, 80], but distinguishing this type of structures from body fossils has been historically problematic[4]. A trace fossil recorded in lowermost Cambrian strata near Sonntagsbrunn and referred to *Curvolithus*[33] is now considered to represent a preservational variant of *T. pedum*[34].

It has been argued that treptichnid trace fossils in the lower part of the Urusis Formation, when compared to the global GSSP, require a lower placement of the Ediacaran–Cambrian boundary in the Nama Group than that commonly used[36, 96]. This lower positioning of the boundary is not consistent with radiometric dating of the base of the Cambrian, currently understood to be close to 541 Ma[31]. Also, based on the limited published material, it remains unclear if these trace fossils are identical to the Cambrian forms or represent similar, but not identical, forms. In the latter case, they are part of the latest Ediacaran trace-fossil zone representing the initial diversification of complex trace fossils.

2. Systematic description

Ichnogenus *Parapsammichnites* n. igen.

Etymology: Ichnogeneric name reflecting similarities with the Phanerozoic ichnogenus *Psammichnites*.

Diagnosis: Mostly horizontal trace fossils with a unilobate to bilobate basal surface, locally displaying thin, transversal lamination organized in inclined sediment pads. Cross sectional view typically flat ellipsoidal to biconvex lenticular. Full reliefs on bed soles, and more rarely, on sandstone tops as positive ridges.

Ichnospecies *Parapsammichnites pretzeliformis* n. isp.

Figs. 2, 3a-g, S6, S7a, b, S8a, b, S9a-g

Etymology: Ichnospecific name from the pretzel-like (i.e. double looping) common configuration.

Horizon and locality: Sandstone unit located in the lower part of the Spitskop Member (Fig. S4), Urusis Formation (Nama Group, Schwarzrand Subgroup), 1.2 km east-southeast of the Koelkrans camp in the Fish River Canyon (Gondwana Canyon Park) region, southern Namibia Koelkrans trace site (GPS latitude-longitude coordinates 27 15 48.4 S 17 42 26.3 E).

Material: The analysed material comprises ten slabs (F730 to F739) housed in the collections of the Earth Sciences Museum, Windhoek, plus extensive material studied in the field, totalling over 60 specimens.

Holotype: Slab number F730 (Fig. 2) curated at the Earth Sciences Museum in Windhoek.

Diagnosis: *Parapsammichnites* forming scribbles, nested loops or spirals, with common self-overcrossing.

Description: Horizontal to subhorizontal trace fossils with a unilobate to bilobate basal surface describing scribbles, circles and, more rarely, spirals and meanders. Self-overcrossing trace fossils commonly form distinctive pretzel-shapes which are well developed on some surfaces (Fig. 3a, c, S9e-g). Width is 0.4-1.1 cm (Fig. S10). Some specimens display significant width changes along the structure revealing different toponomic levels or burrowing strategies (Fig. 3c). Penetration depth is at least 1 cm. Specimens display a unilobate external morphology that is not totally continuous, but may show some constrictions, in places displaying inclined, slightly imbricated arcuate ridges or sediment pads. Some specimens are composed of bilobate segments (Fig. 3b, c-e, S9a, b). Bilobate structures seem to record either an internal morphology as suggested by some specimens (Fig. 3d) or alternatively may record a change in locomotion style. Arcuate sediment pads exhibit locally an imbricated morphology and show a slight offset from

the axis position (Fig. 3e). Shorter sediment pads are more closely spaced and result in a roughly annulated aspect (Fig. 3d). Some strongly weathered specimens display no relief, but clearly reveal the internal thin laminae of the backfill (Fig. 3g). An irregular lobular zone of disturbed sediment (Fig. 3c) is observed in some specimens that show a significant increase in width along the structure. This envelope of disturbed sediment results in an irregular and more diffuse burrow boundary. Preserved as full reliefs on sandstone bases or, more rarely, on tops (as epichnial ridges).

3. Ichnologic Remarks

Parapsammichnites pretzeliformis belongs to a class of ichnofossils that represent continuous movement within sandy sediments parallel to the sea-floor, forming irregular curves, circles or loops. Comparable lower Cambrian trace-fossil specimens have a complex internal and external morphology showing one or several of the following features: a bilobed and/or unilobed basal and/or internal surface, transverse or lunate markings on the burrow wall corresponding to sediment manipulation with resulting back-fill and/or muscular activity in burrow formation, and an upper raised bilobed surface formed by sediment displacement that is bisected by a sinusoidal or straight furrow[56, 97, 98]. The furrow has been suggested to represent the trace of a siphon of snorkel-type organ[30]. This type of trace fossil has been variously referred to the ichnogenera *Psammichnites*, *Plagiogmus* and *Didymaulichnus*, but also to ichnospecies of *Taphrhelminthopsis*[56, 97-101]. The morphology observed in each individual occurrence depends strongly on the levels within the complex burrow structure that are exposed to view as well as on preservational conditions, and this has led to a difference of opinion as to whether these forms

are all best included in *Psammichnites* or better maintained as separate ichnogenera.

Certain late Ediacaran trace fossils probably represent the same general behaviour as seen in *Psammichnites*. These are of smaller dimensions than the Cambrian forms, and do not show the same range of morphological features, either because these were never there or because their smaller size did not permit their preservation. Ediacaran trace fossils of this type have been referred to as *Aulichnites* and *Archaeonassa* and probably also include some of the trace fossils that have been included in *Helminthoidichnites*. *Psammichnites*-type trace fossils, having the possible imprint of a siphon, have been recorded in the Ediacaran of the White Sea area[102]. The internal complexity revealed in *Parapsammichnites pretzeliformis* by sections exposed at several preservational levels, in addition to the grazing pattern, is hitherto unknown in the late Ediacaran, underscoring the evolutionary significance of these structures. The level of morphologic and ethologic complexity of *Parapsammichnites pretzeliformis* is comparable to that of trace fossils reported as *Didymaulichnus* and in particular "*Taphrhelminthopsis*" *circularis* from basal Cambrian strata.

4. Sedimentologic description and interpretation of the trace fossil-bearing deposits

The trace fossils are preserved at the base and, more rarely, at the top of 0.5-1 cm thick, sharp-based, parallel-laminated and current-ripple cross-laminated, micaceous, very fine-grained silty sandstone beds. The sandstone bases are locally covered by tool marks and primary current lineation. Thin lenses of subangular to well-rounded mudstone intraclasts up to 6 cm across commonly mantle the sandstone bases (SI Appendix, Fig. S5a). Prod marks and narrow to broad, comb-like tool marks ascribed to vendobionts are common (SI Appendix, Fig. S5b). The trace

fossil-bearing sandstone layers are stacked forming a 30 cm-thick bedset. This bedset passes upwards into a 2.6 m thick, cross-bedded, fine- to very fine-grained (locally up to medium-grained) silty sandstone unit with straight-crested dunes (wavelength 1.1-2.6 m, amplitude 6-16 cm) preserved at the top (SI Appendix, Fig. S5c). Internally, the sandstone displays cross bedding with tangential basal contacts, gently inclined reactivation surfaces, scours (SI Appendix, Fig. S5d), and thin mudstone intraclast layers. Paleocurrent measurements indicate currents toward the south/south-west. Three types of ripples are superimposed to the dunes: (1) subordinate sinuous-crested ripples on the dune lee face (wavelength 7-13 cm, amplitude 0.5-1.0 cm) (SI Appendix, Fig. S5e), (2) straight-crested ripples oriented perpendicular to the dune crest on the stoss side (wavelength 4-5 cm, amplitude 0.2-0.3 cm), and (3) downslope-migrating, slightly sinuous-crested ripples on the dune lee face (wavelength 3-12 cm, amplitude 0.4-0.5 cm). This unit passes upward into a 40-cm thick, current-ripple cross-laminated, very fine-grained silty sandstone with interference ripples (SI Appendix, Fig. S5f) and syneresis cracks (of Manchuriophycus / Rhysonetron type) (SI Appendix, Fig. S5g).

The presence of tool marks, primary current lineation and intraclasts in the trace-fossil bearing beds evinces strong erosion in the deposit. Most of the trace fossils cross-cut the inorganic tool marks, indicating a post-depositional origin and penetration from the top of the sandstone layer (burrowing depth up to at least 1 cm). Washed-out specimens suggest erosion of the overlying sandstone layer and casting of pre-existing subsurface burrows. The internal structure of the sandstone beds indicates unidirectional currents. Evidence of oscillatory flows has not been recorded. The presence of reactivation surfaces mantled by subordinate current ripples in the overlying bedsets indicates that these are tide-generated structures formed under a strong subordinate current capable of eroding the lee face[103-105]. These deposits are interpreted as

formed in shallow-subtidal small dunes. In particular, the trace-fossil bearing beds represent the bottomsets of the dune system. The presence of interference ripples at the top of the sandstone unit suggests shallower-water conditions during dune abandonment.

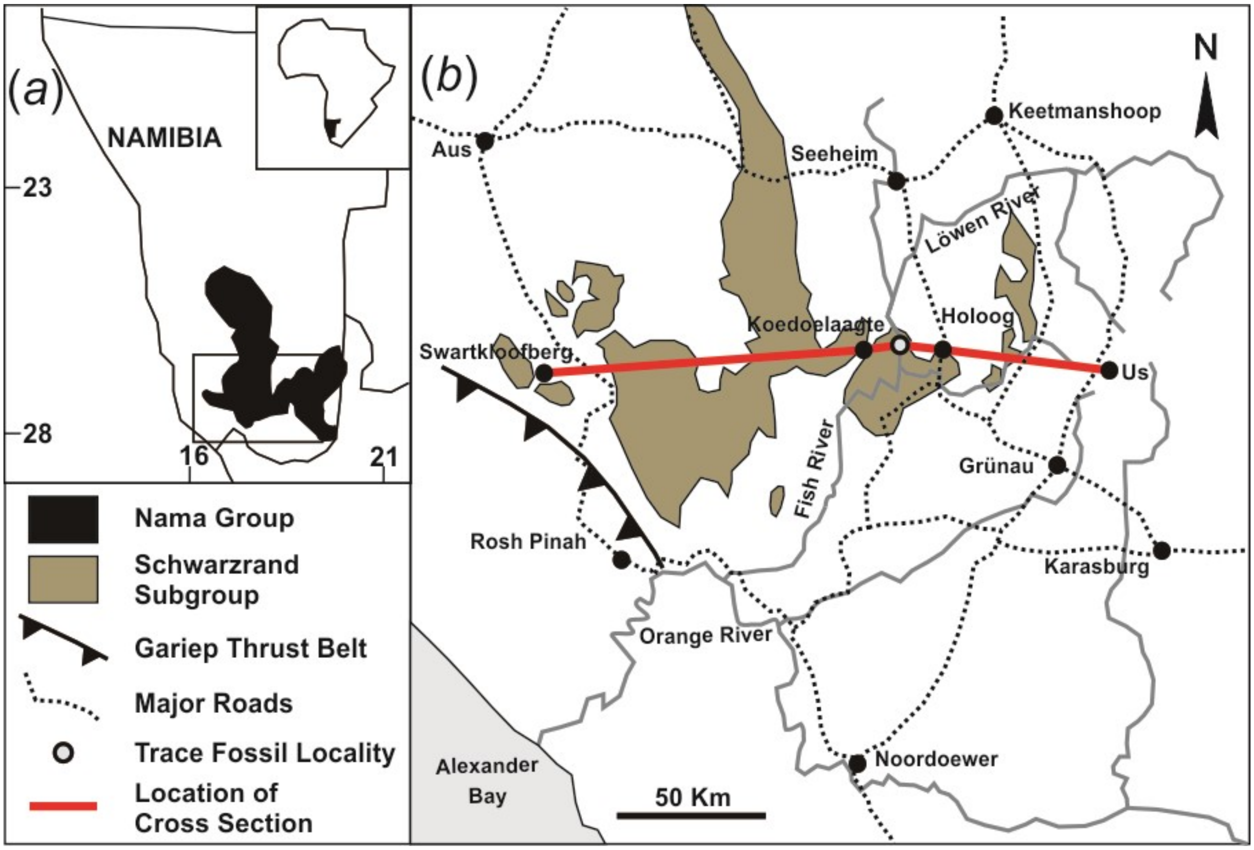

Figure S1. Location maps. **(a)** General map outlining the distribution of the Nama Group in Namibia. **(b)** Map showing the distribution of the Schwarzrand Group in the Witputs sub-basin and the location of the trace-fossil site in Fish River Canyon / Gondwana Canyon Park region. Co-ordinates of the trace-fossil site (acquired with GPS) are 27º 15’ 48.4” S, 17º 42’ 26.3” E (maps designed and drawn by Gerard J. B. Germs and John Almond using Corel DRAW X4 software).

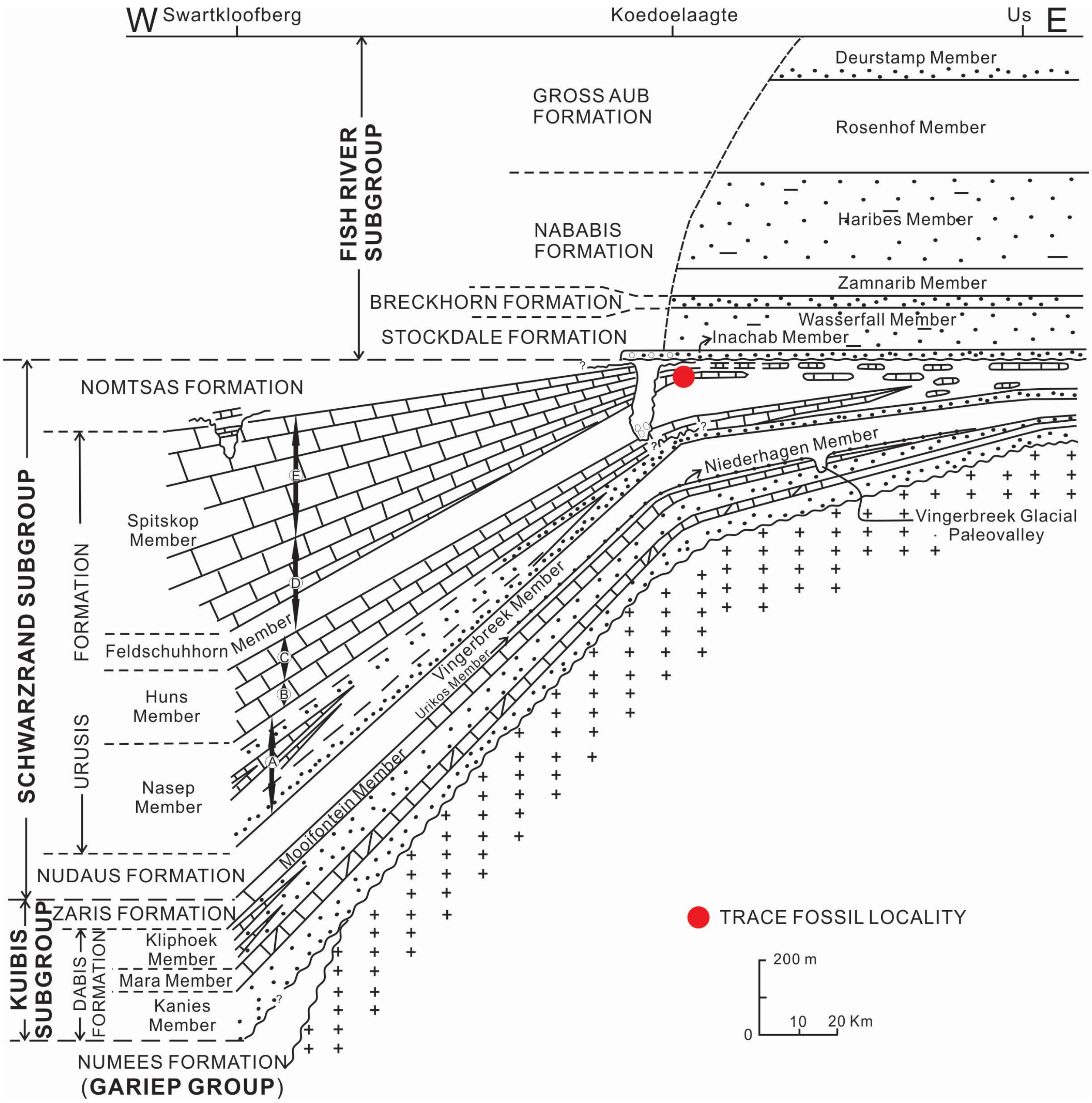

Figure S2. E-W stratigraphic cross-section of the Nama Group showing lateral distribution of the different lithostratigraphic units and the location of the trace-fossil site (redrawn based on figure 4 from Ref. 42 and designed by Gerard J. B. Germs, John Almond and Luis A. Buatois using Corel DRAW X4 software; permission by the Geological Society of South Africa). See Supplementary Figure 1 for location of the cross-section. Clastic intercalations within the Huns and Spitskop Members are not shown. Depositional sequence code (A-E) after Ref. 41.

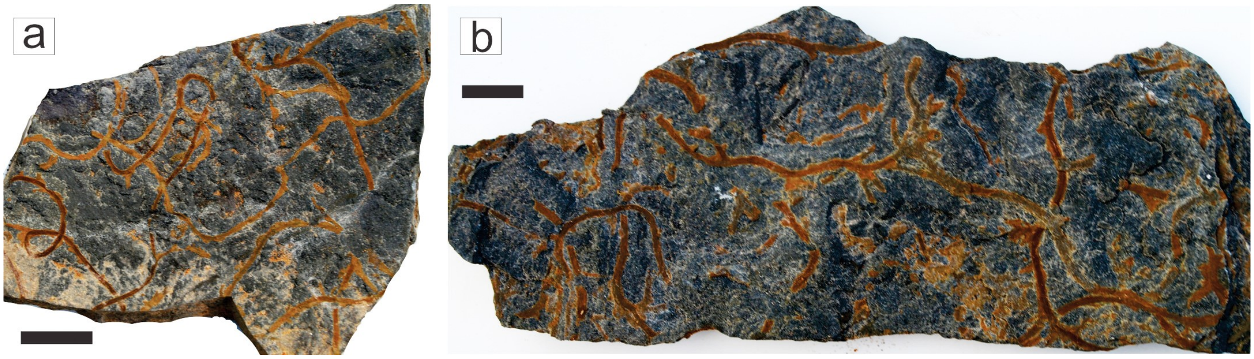

Figure S3. The vendotaenid *Vendotaenia antiqua* in the Spitskop Member at Koelkrans. Scale bars are 1 cm wide. **(a)** Various tubes showing sinuous bending and overcrossing. **(b)** Sinuous specimens showing branching.

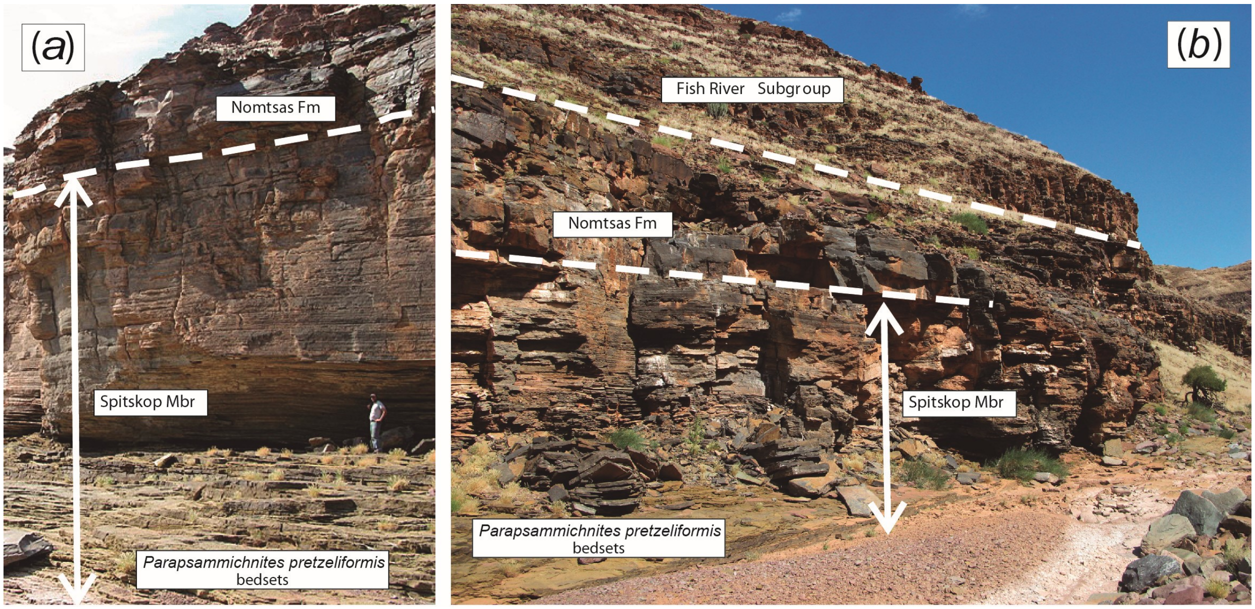

Figure S4. (a) (b) General view of the studied outcrop. Person (1.75 m high) standing on top of the trace fossil-bearing deposits in **(a)**.

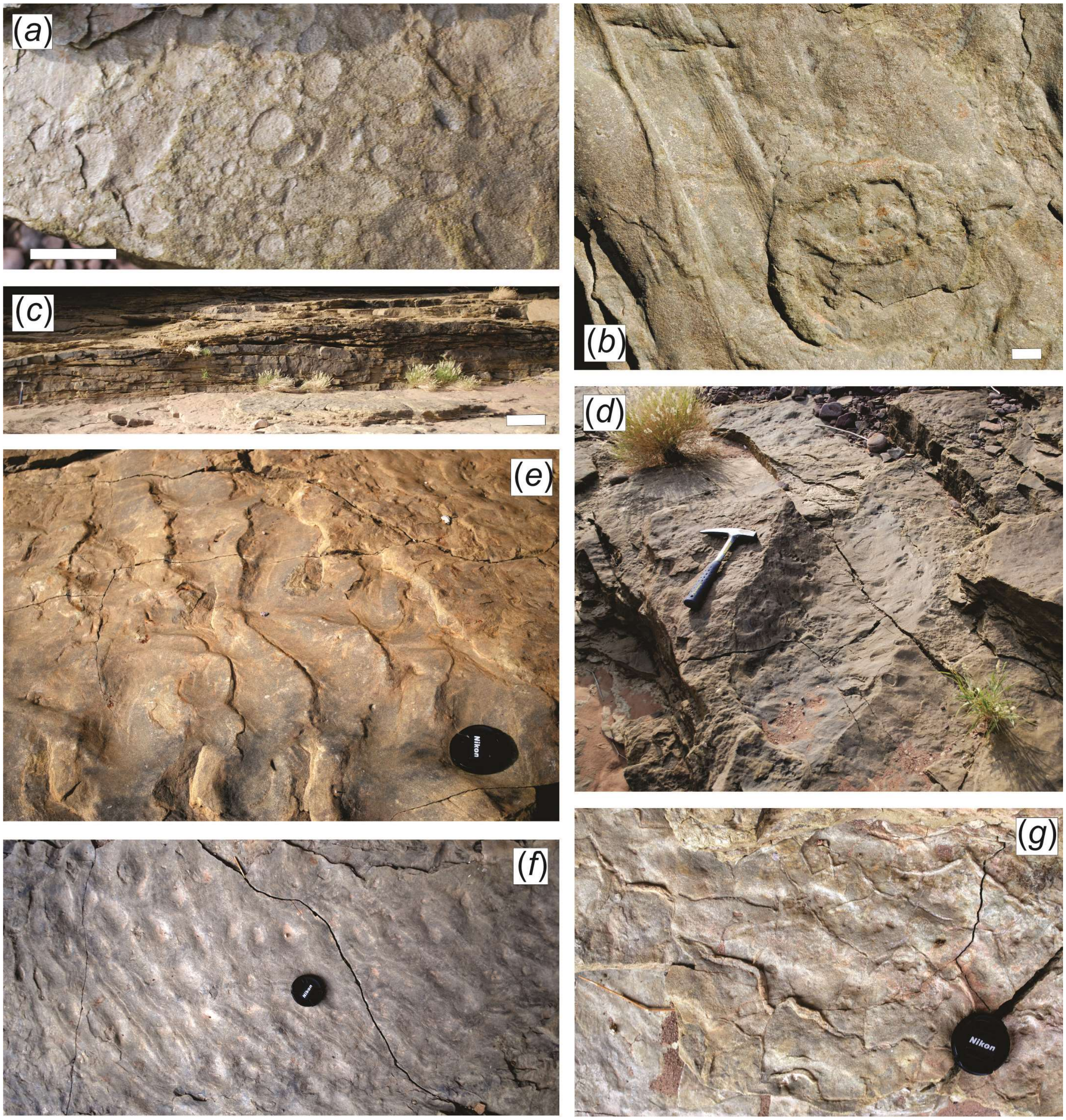

Figure S5. Sedimentological features of the trace-fossil bearing deposits and associated facies. **(a)** Mudstone intraclasts forming a thin lens at the base of the trace fossil-bearing sandstone bedset. Scale bar is 5 cm. **(b)** Broad comb-like tool marks, probably produced by vendobiontans, which are cross-cut by later trace fossil. Scale bar is 1 cm. **(c)** General view of the cross-bedded sandstone unit overlying the trace fossil-bearing bedset. Scale bar is 1 m. **(d)** Relatively large scour on cross-bedded sandstone unit (length of hammer is 34 cm). **(e)** Subordinate sinuous-crested ripples on the dune lee face. Lens cover is 5.5 cm wide. **(f)** Interference ripples at the top of the unit. Lens cover is 5.5 cm wide. **(g)** Syneresis cracks at the same level as the uppermost interference ripples. Lens cover is 5.5 cm wide.

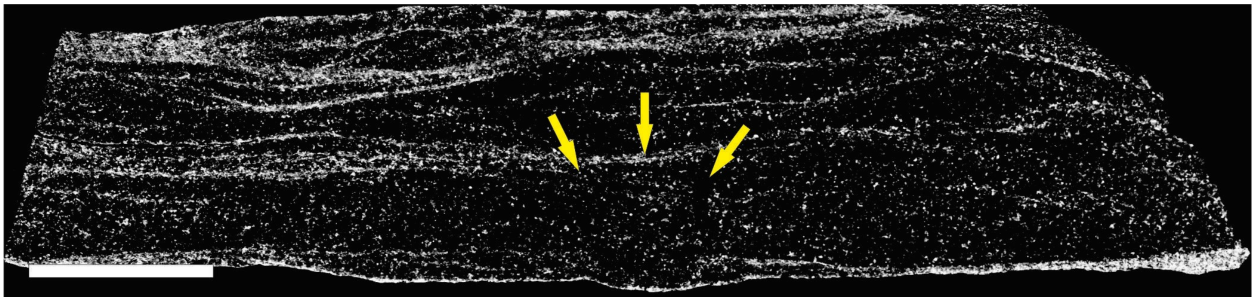

Figure S6. Polished cross-section view of slab containing *Parapsammichnites pretzeliformis* (arrows) cross-cutting of the primary fabric in the lowermost layer. The trace fossil penetrates down to the lithologic interface, demonstrating its infaunal nature. Note the absence of cross-lamination near the burrow and the undisturbed ripple cross-lamination preserved in the overlying unbioturbated layer. This style of sediment penetration is remarkably similar to that shown by the more complex Cambrian *Psammichnites gigas* (cf. fig. 4b in Ref. 98).

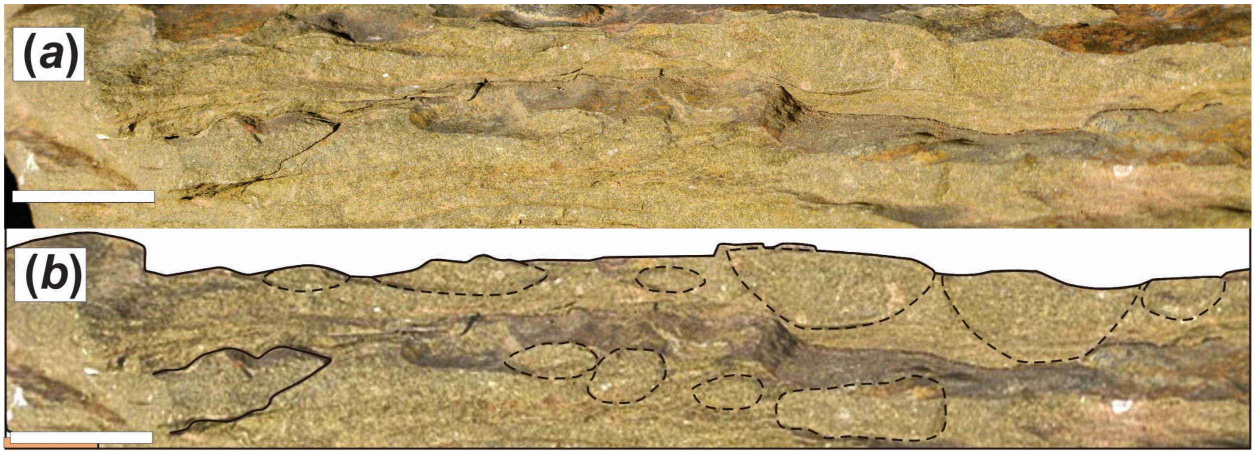

Figure S7. Cross-section view of *Parapsammichnites pretzeliformis* showing disturbance of the primary fabric. **(a)** Photograph. **(b)** With line drawing overlay. Scale bar is 1 cm.

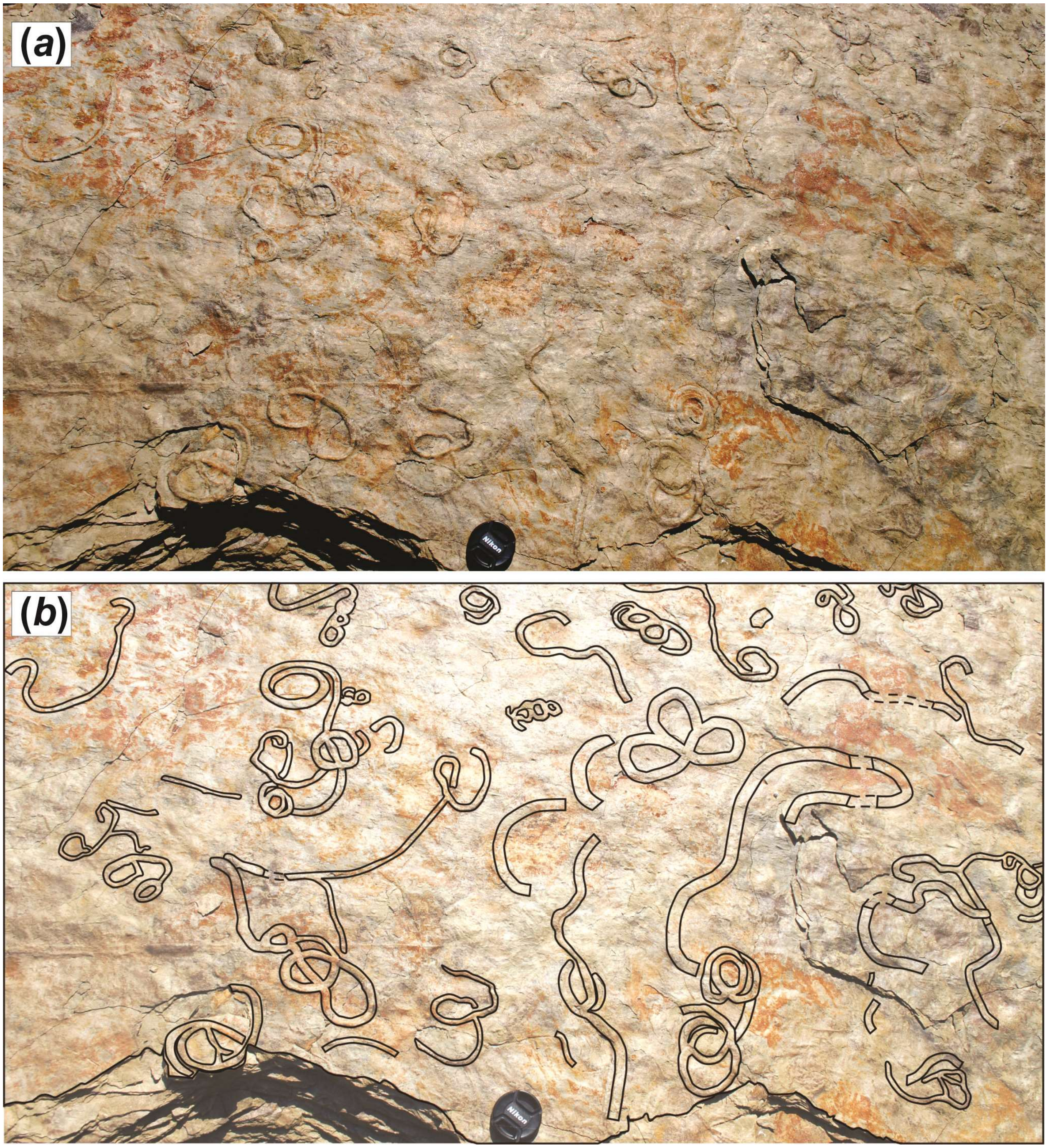

Figure S8. Density of *Parapsammichnites pretzeliformis* on bedding surfaces. (a) Photograph. (b) Line drawing. Scale bar is 1 cm. *Parapsammichnites pretzeliformis* is observed to cross through mm-thick lamina on this surface.

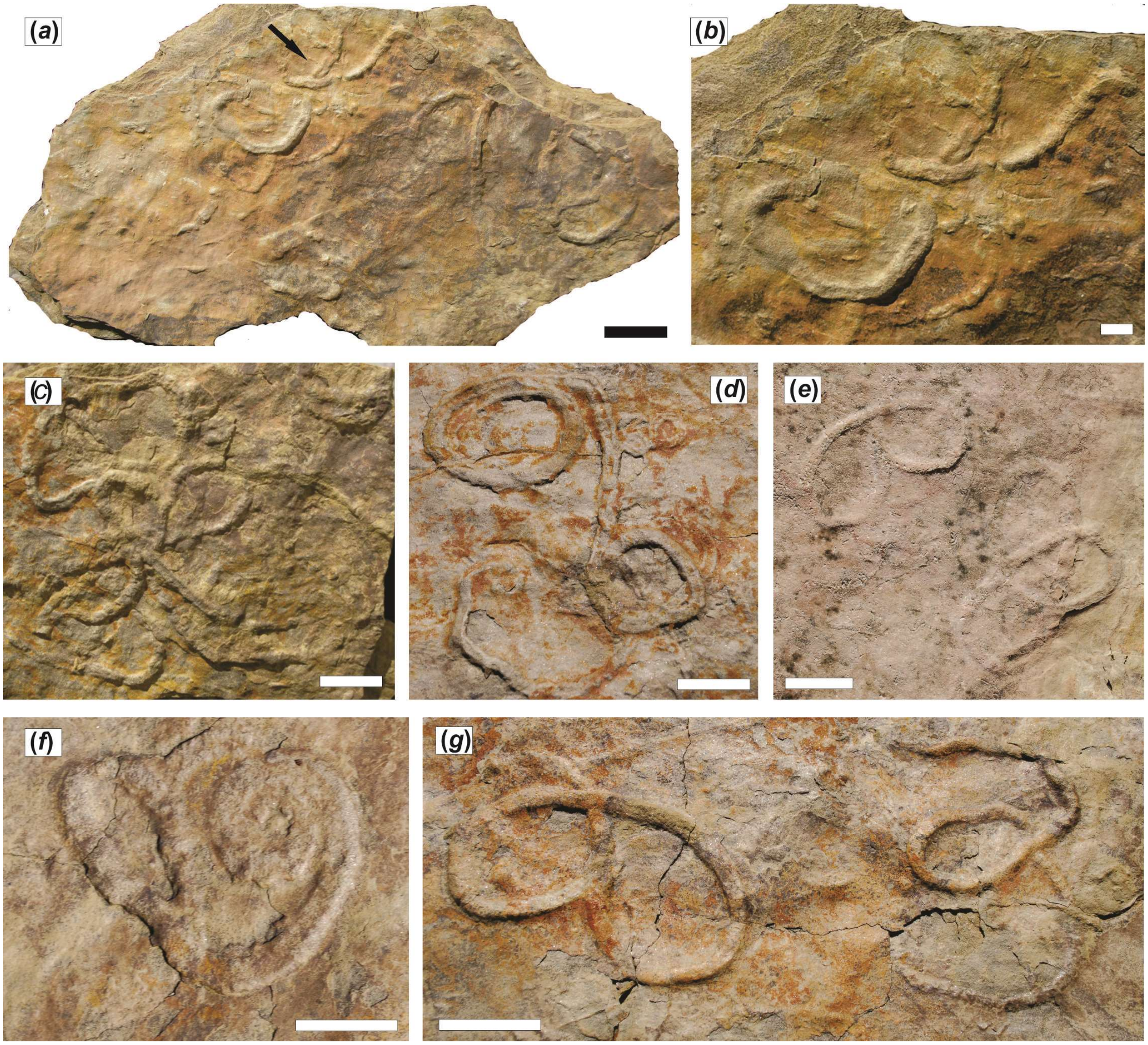

Figure S9. Morphologic variability of *Parapsammichnites pretzeliformis*. **(a)** General view of a slab containing several specimens displaying self-overcrossing. **(b)** Close-up of one of the specimens illustrated in (a). Note change from bilobate to unilobate basal morphology along specimen. **(c)** Long specimen displaying multiple self-overcrossing and tortuous scribbling course **(d)** Specimen developing a double spiral course (spirals in opposite directions mimicking the trace fossil *Spirodesmos bicornis*). **(e)** Two typical "pretzel-like specimens preserved as convex epirelief. **(f)** Combined pretzel-like and spiral (right arm) configuration. **(g)** Two specimens showing classic pretzel (left) and loop (right) courses. Note specimen on the right can be traced into lower laminae. All views are from bed bases, with the exception of (e) and (g), which are top views. All scale bars are 5 cm, with the exception of (b), which is 1 cm.

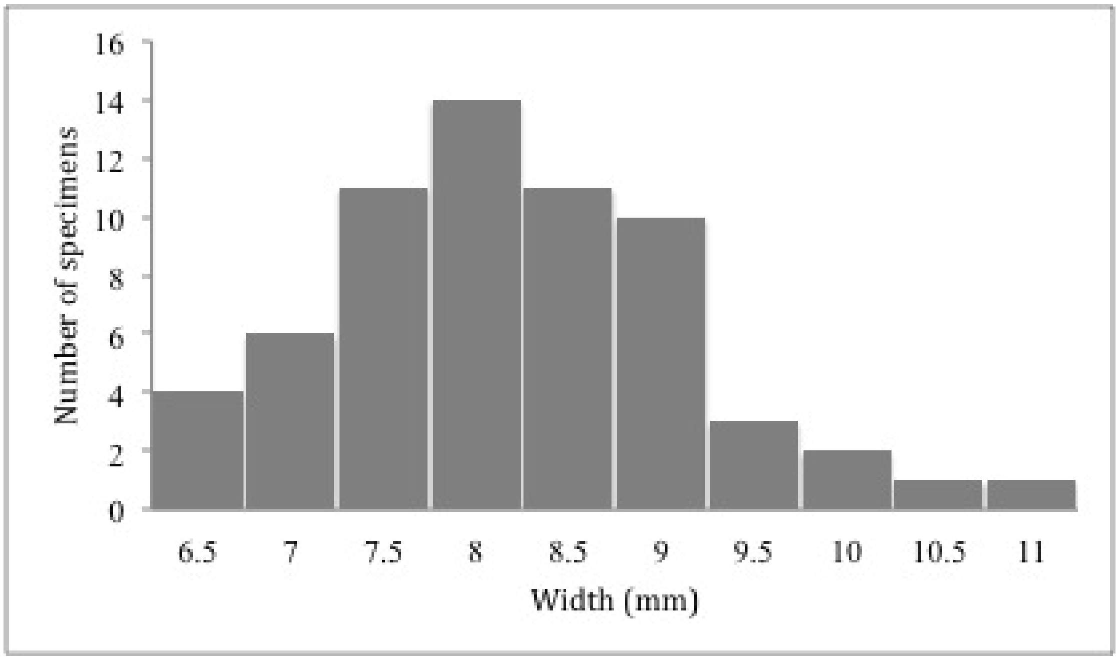

Figure S10. Histogram showing size (width) distribution of the studied trace fossils. n = 67.

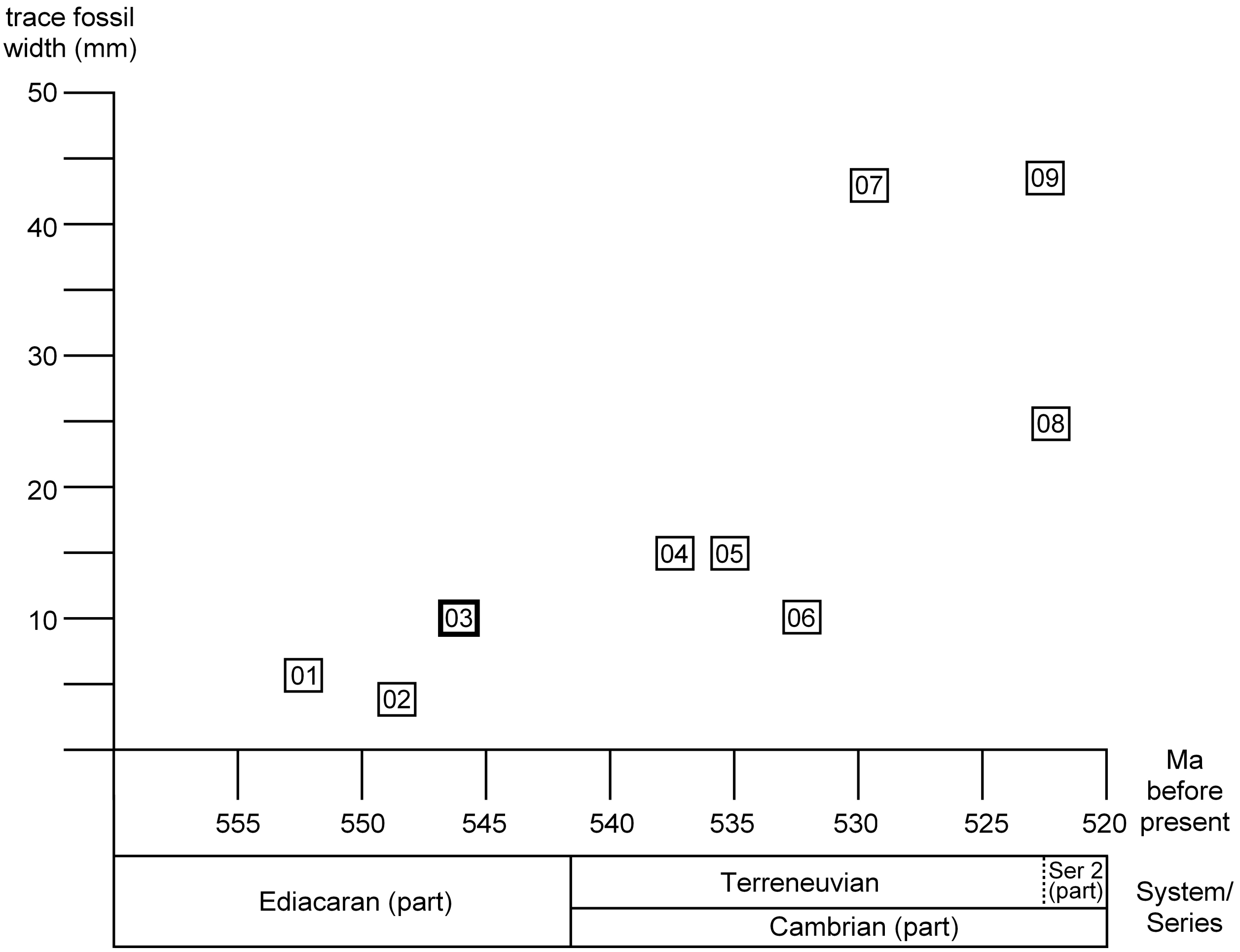

Figure S11. Changes in size of bulldozer-type trace fossils, as a proxy for body-size of the producers, across the Ediacaran-Cambrian transition. Width of trace fossil is a proxy for the width of the bulldozer producer. Plot is limited to occurrences for which there exists age control better than ca 5 Ma. 1, *Aulichnites* isp., Zimnegory Formation, northern Russia [106]; 2, *Psammichnites* isp., Zigan Formation, Urals region, Russia [102]; 3, this study; 4, "*Taphrhelminthopsis" circularis*, Chapel Island Formation, Newfoundland, Canada [107]; 5, *Didymaulichnus miettensis*, Kessyusa Formation, northern Siberia [97]; 6, *Didymaulichnus miettensis*, south China [108]; 7, *Psammichnites gigas*, Ratcliffe Brook Formation, New Brunswick, Canada [98]; 8, *Psammichnites gigas*, Hardeberga sandstone, Sweden [109]; 9, *Psammichnites gigas*, Marcory Formation, France [110]. The trace fossils described in this study are comparable in size to traces from the Phanerozoic Era and significantly larger than any previously recorded from Precambrian rocks.

References

93. Germs, G. J. B. Trace fossils from the Nama Group, south-west Africa. *J. Paleontol*. **46**, 864–870 (1972).
94. Bouougri, E. H. & Porada, H. Siliciclastic biolaminites indicative of widespread microbial mats in the Neoproterozoic Nama Group of Namibia. *J. Afr. Earth Sci.* **48**, 38–48 (2007).
95. Macdonald, F. A., Pruss, S. B. & Strauss, J. V. Trace fossils with spreiten from the late Ediacaran Nama Group, Namibia: Complex feeding patterns five million years before the Precambrian–Cambrian boundary. *J. Paleontol.* **88**, 299–308 (2014).
96. Geyer, G. The Fish River Subgroup in Namibia: Stratigraphy, depositional environments and the Proterozoic–Cambrian boundary problem revisited. *Geol. Mag.* **142**, 465–498 (2005).
97. Dzik, J. Behavioral and anatomical unity of the earliest burrowing animals and the cause of the "Cambrian explosion". *Paleobiology* **31**, 503–521 (2005).
98. Hofmann, H. J. & Patel, I. M. Trace fossils from the type 'Etcheminian Series' (Lower Cambrian Ratcliffe Brook Formation), Saint John area, New Brunswick, Canada. *Geol. Mag.* **126***,* 139–157 (1989).
99. Crimes, T. P., Legg, I., Marcos, A. & Arboleya, M. In Trace fossils 2 (eds Crimes, T. P. & Harper, J. C.). *Geol. J. Spec. Iss.* **9**, 91–138 (1977).
100. McIlroy, D. & Heys, G. R. Palaeobiological significance of *Plagiogmus arcuatus* from the lower Cambrian of central Australia. *Alcheringa* **21**, 161–178 (1997).
101. Hagadorn, J. W., Schellenberg, S. A. & Bottjer, D. J. Palaecology of a large Early Cambrian bioturbator. *Lethaia* **33***,* 142–156 (2000).

102. Grazhdankin, D. D., Marusin, V. V., Meertb, J., Krupeninc, M. T. & Maslovc, H. V. Kotlin Regional Stage in the South Urals. *Dokl. Earth Sci.* **440**, 1222–1226 (2011).

103. Visser, M. J. Neap-spring cycles reflected in Holocene subtidal large-scale bedform deposits: a preliminary note. *Geology* **11**, 543–546 (1980).

104. Dalrymple, R. W. In *Facies Models 4 GEOtext 6* (eds James, N. P. & Dalrymple, R. W.) 233-264 (GAC, 2010).

105. Desjardins, P. R., Buatois, L. A., Pratt, B. R. & Mángano, M. G. Subtidal sandbody architecture and ichnology in the Early Cambrian Gog Group of western Canada: Implications for an integrated sedimentologic-ichnologic model of tide-dominated shelf settings. *Sedimentology* **59**, 1452–1477 (2012).

106. Fedonkin, M. A. Iskopaemye sledy dokembrijskikh metazoa. *Izvestia Akad. Nauk. SSSR, Ser. Geol.* **1980**, 39–46 (1980).

107. Narbonne, G. M., Myrow, P. M. & Anderson, M. M. A candidate stratotype for the Precambrian–Cambrian boundary, Fortune Head, Burin Peninsula, southeastern Newfoundland. *Can. J. Earth Sci.* **24**, 1277–1293 (1987).

108. Crimes, T. P. & Jiang, Z. Trace fossils from the Precambrian–Cambrian boundary candidate at Meishucun, Jinning, Yunnan, China. *Geol. Mag.* **123**, 641–649 (1986).

109. Torell, O. Petrificata suecana formationis cambricae. *Lunds Univ. Årsskrift* **8**, 1–14 (1870).

110. Álvaro, J. J. & Vizcaïno, D. Biostratigraphic significance and environmental setting of the trace fossil *Psammichnites* in the Lower Cambrian of the Montagne Noire, France. *Bull. Soc. Geol. France* **170**, 821–828 (1999).
